# Circulating tumor DNA (ctDNA) trajectories predict survival in trifluridine/tipiracil‐treated metastatic colorectal cancer patients

**DOI:** 10.1002/1878-0261.13755

**Published:** 2025-01-22

**Authors:** Matthias Unseld, Stefan Kühberger, Ricarda Graf, Christine Beichler, Markus Braun, Nadia Dandachi, Ellen Heitzer, Gerald W. Prager

**Affiliations:** ^1^ Division of Palliative Care, Department of Medicine I Medical University of Vienna Austria; ^2^ Institute of Human Genetics, Diagnostic and Research Center for Molecular BioMedicine Medical University of Graz Austria; ^3^ Christian Doppler Laboratory for Liquid Biopsies for Early Detection of Cancer Medical University of Graz Austria; ^4^ Division of Oncology, Department of Internal Medicine Medical University of Graz Austria; ^5^ Research Unit for Epigenetic and Genetic Cancer Biomarkers Medical University of Graz Austria; ^6^ Division of Oncology, Department of Medicine I Medical University of Vienna Austria

**Keywords:** circulating tumor DNA, joint model, metastatic colorectal cancer, mixed model, molecular profiling, trifluridine/tipiracil

## Abstract

Late‐line treatment in metastatic colorectal cancer (mCRC) can improve prognosis. However, not every patient has a benefit and may experience severe side effects. Thus, predictive/prognostic biomarkers are urgently needed. We investigated the prognostic role of circulating tumor DNA (ctDNA) in mCRC patients before and during treatment with trifluridine/tipiracil (FTD/TPI). This noninterventional translational biomarker phase II study enrolled 30 mCRC patients (60% male, 40% female). Using a 77‐gene panel, ctDNA was detectable in 90% of patients. Tumor levels were assessed based on aneuploidy (ichorCNA) as well as the highest variant allele frequency, and correlated with overall survival (OS). Uni‐ and multivariate survival analyses were performed with clinical variables. Longitudinal changes in tumor levels over time were analyzed with linear mixed and joint models. The median OS was 8.1 months, with a recorded disease control rate of 30%. High ctDNA levels (≥ 5%) were associated with inferior survival after undergoing FTD/TPI therapy. Elevated tumor level trajectories over time were associated with higher risks of death. Therefore, ctDNA can help identify patients who are unlikely to benefit significantly from this treatment in late‐stage disease, thus preventing unnecessary treatments and their associated side effects, ultimately enhancing quality of life.

## Graphical abstract

The authors applied joint/mixed models that predict mortality of trifluridine/tipiracil‐treated metastatic colorectal cancer patients based on circulating tumor DNA (ctDNA) trajectories. Patients at high risk of death could be spared aggressive therapy with the prospect of a higher quality of life in their remaining lifetime, whereas patients with a good prognosis may benefit from further treatment.

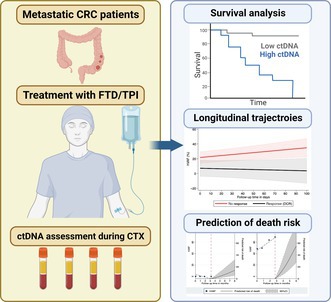

**Abbreviations**
AIC: Akaike's information criterion
aMM: average mutant molecules per mL plasma
aVAF: average variant allele frequencies
BCT: blood collection tubes
BL: baseline
ctDNA: circulating tumor DNA
ECOG: Eastern Cooperative Oncology Group
FTD/TPI: trifluridine/tipiracil
FU: follow‐up
hMM: highest mutant molecules per mL plasma
hVAF: highest variant allele frequencies
iTF: ichorCNA tumor fraction
mCRC: metastatic colorectal cancer
MM: mutant molecules per mL plasma
NCCN: National Comprehensive Cancer Network
OS: overall survival
PD: progressive disease
PFS: progression‐free survival
PR: partial response
SD: stable disease
sWGS: shallow whole‐genome sequencing
VAF: variant allele frequencies

## 1 Introduction

Despite improvements in diagnosis and treatment, metastatic colorectal cancer (mCRC) is still among the top five leading causes of cancer death worldwide [1]. However, new treatment options, including targeted treatment against VEGF(R) or EGFR as well as immunotherapy, have prolonged overall survival (OS) of metastatic patients to 30 months and more [2, 3, 4]. One option for cytotoxic treatment is the oral nucleoside compound trifluridine/tipiracil (FTD/TPI), which inhibits cellular proliferation and causes cell death by incorporation of its triphosphorylated thymidine‐based nucleoside form into the DNA [5]. In the RECOURSE trial, FTD/TPI was demonstrated to significantly prolong OS (7.1 vs. 5.3 months, respectively; HR, 0.58; *P* < 0.001) and progression‐free survival (PFS) (2.0 vs. 1.7 months, respectively; HR, 0.48; *P* < 0.001) when compared to placebo in patients with chemorefractory mCRC [6]. Based on these results, FTD/TPI has been approved as a third‐line treatment in patients with refractory mCRC by both the FDA and the EMA, respectively [7]. Yet, efficacy is limited to a median PFS of only 2 months. In addition, despite a disease control rate (DCR) of around 44%, a significant number of patients experienced adverse events, such as hematological side effects of grade 3, nausea and loss of appetite, thereby impacting the quality of life [5]. The SUNLIGHT trial demonstrated that treatment with FTD‐TPI plus bevacizumab can extend survival [8].

While clinical markers such as two or fewer metastatic sites, no liver metastasis, or an indolent disease (time from first diagnosis > 18 months) are predictive of long‐term survival, molecular markers to identify patients who will benefit from FTD/TPI or predict survival have not been described yet.

In the last decade, cell‐free circulating tumor DNA (ctDNA) in plasma evolved as a promising predictor of risk stratification for recurrence and to monitor therapy outcomes [9, 10]. Due to its short half‐life, ctDNA may more accurately reflect treatment response than conventional tumor markers for patients with mCRC, and some studies have already demonstrated superiority over standard tumor markers [11, 12, 13]. ctDNA is detectable in a high number of mCRC and several studies have investigated its potential as a predictor of treatment response or survival [11, 12, 14]. For example, in the ongoing phase 3 ALTAIR trial, a Japanese study group aims to establish the superiority of FTD/TPI compared with placebo in patients with resected CRC who show molecular residual disease after ctDNA testing at any time after curative resection [15]. However, ctDNA as a possible response parameter or predictor of long‐term FTD/TPI treatment outcome has not been described so far.

In this prospective exploratory study, we investigated ctDNA as a prognostic and/or predictive marker for mCRC patients receiving FTD/TPI. To this end, we integrated different sequencing approaches, including shallow whole‐genome sequencing (sWGS) and a gene panel to longitudinally assess ctDNA levels and correlate them with treatment response and survival.

## 2 Materials and methods

### 2.1 Study design

This translational exploratory study was performed at the Division of Oncology, Department of Medicine I of the Medical University Vienna (Austria). Patients with histologically proven mCRC receiving FTD/TPI therapy between 12/2018 and 07/2020 were enrolled. Patients received FTD/TPI in standard dose (35 mg·m^−2^ twice daily on days 1–5 and days 8–12 in a 28‐day cycle) in concordance with its label plus best supportive care. Patients with active or clinically significant cardiac disease, thrombotic, embolic, venous, or arterial events, such as cerebrovascular accident (including transient ischemic attacks), deep vein thrombosis or pulmonary embolism within 6 months of informed consent, and previous malignancy other than CRC in the last 3 years, were excluded. Adverse events were reported to the authorities according to legal regulations but were not subject to this analysis. CT scans of the chest and abdomen were performed at baseline (BL) and then every 8 weeks during treatment (or earlier for patients with suspected disease progression). CT scans were centrally reviewed to evaluate treatment response according to RECIST criteria, version 1.1.

This study (NCT01983098) received ethical approval from the institutional ethics committee of the Medical University of Vienna (EK number 1992/2017) and was carried out in accordance with the requirements of the International Conference on Harmonization E6 for Good Clinical Practice as laid down in the Helsinki Declaration. All patients provided written informed consent.

### 2.2 Blood collection and cfDNA extraction

Blood was collected at the time of treatment initiation (BL) and during follow‐up after an average of 4 weeks (FU1; range, 2.7–7.5), 8.5 weeks (FU2; range, 4.3–13.6), and 12.3 weeks (FU3; range, 8.0–15.8) in cell‐free DNA blood collection tubes (BCTs) (Streck, Omaha, NE, USA). Cell‐free DNA BCTs were processed within 24 h according to the manufacturer's instructions (Double Spin Protocol 2). After a double spin, the supernatants were transferred in 2 mL fractions and stored at −80 °C until cell‐free DNA extraction [16]. cfDNA was isolated from 2 mL of plasma using the QIAamp Circulating Nucleic Acid Kit (QIAGEN, Hilden, Germany). Quantification of cfDNA was performed using the Qubit dsDNA HS Assay Kit (Thermo Fisher Scientific, Vienna, Austria).

### 2.3 Molecular profiling of plasma samples

Molecular profiling was performed using the AVENIO ctDNA Expanded Kit (Roche, Basel, Switzerland). This panel consists of 77 genes, including genes currently described in the U.S. National Comprehensive Cancer Network (NCCN) Guidelines as well as emerging biomarkers currently being investigated in clinical trials (Table S1). In‐house validation of the assay confirmed the vendor's specification [17]. AVENIO libraries were prepared from an average of 40 ng cfDNA (range, 14–50 ng) in accordance with the manufacturer's instructions. Libraries were quantified using Qubit dsDNA HS Assay Kit and pooled equimolarly. Pools were quantified using qPCR and sequencing was performed in pools of 4 or 11 in a 150PE mode on an Illumina NextSeq Mid or High Output kit, respectively, generating an average of 30 million read pairs for the cfDNA samples (range, 15–48 million). After consensus read generation, this resulted in an average read depth of 4340× (range, 1664–8237×) and a median fragment length of 175 bp (range, 161–317 bp). Data were analyzed using the avenio oncology analysis Software, version 1.1.0 (Roche), with customized somatic variant filtration settings. Variants commonly found in germline defined by GnomAD v2.1 with MAF ≥ 1% or common variants listed in dbSNP155 were filtered. Moreover, ctDNA variants that were unique to a single time point and covered by less than 10 alternative reads were excluded. In contrast, variants observed at more than one time point were kept regardless of the number of mutated reads. The sequencing coverage and quality statistics for each sample are summarized in Table S2.

### 2.4 Assessment of ctDNA levels

ctDNA levels were assessed as variant allele frequencies (VAF, percentage of sequence reads observed matching a specific DNA variant divided by the overall coverage at that locus) and the number of mutant molecules per ml plasma (MM). For both measures, we used the average of all mutations per patient (aVAF, aMM) and the mutation with the highest value (hVAF, hMM) (if more than one mutation was identified, the highest allelic fraction or number of MM at BL was used; if only one mutation was observed average equals highest). In addition, we performed sWGS to estimate the tumor fraction (iTF) independent of mutations using the ichorCNA algorithm, a probabilistic HMM model for estimating tumor fraction, roughly equivalent to tumor purity from bulk tumor analyses [18]. To this end, unenriched AVENIO libraries were sequenced on an Illumina MiSeq or NextSeq in a 75 bp PE run obtaining an average of 1.1 million read pairs (range, 0.6–2.6). The sequencing coverage and quality statistics for each sample are summarized in Table S3.

### 2.5 Statistics

Descriptive statistics were used for patient and tumor characteristics with median and range for continuous variables, and frequency and percentage for categorical variables. The Mann–Whitney test was used to compare ctDNA levels between response groups (partial response, PR, and stable disease, SD, vs. progressive disease, PD). Correlations between the various proxies for ctDNA levels (aMM, hMM, aVAF, hVAF, and iTF) were calculated using Spearman correlation coefficients. Median follow‐up time was estimated using the reverse Kaplan–Meier estimator [19]. To assess the effect of ctDNA levels on survival, Kaplan–Meier method and log‐rank test were used. For the clinically most relevant time points (BL and FU1), patients were stratified based on various hVAF and iTF cut‐offs ranging from 1% to 15% to identify the most discriminative cut‐off. OS was defined from the start of treatment (BL) until death or the date of last follow‐up (censored data). Moreover, OS analysis at FU1 was performed, which may represent a critical time point for clinicians in deciding whether to continue the treatment. Hazard ratios (< 1 indicates shorter survival) and *P*‐values were provided for the index variables. Forest plot was used to visualize the estimated hazard ratios. In all analyses, a *P*‐value of 0.05 or smaller was considered significant. Data were analyzed and visualized using graphpad prism (boston, ma, usa). Univariate cox proportional hazards analysis was used to examine whether clinical factors (age, sex, Eastern Cooperative Oncology Group – ECOG, line of treatment, number of metastases, LDH, CRP, CA19‐9) are associated with survival. Factors with *P* < 0.05 as well as factors that were previously reported to correlate with OS (ECOG and number of metastases) were included in a multivariate model.

Longitudinal changes in hVAF levels and iTF over time were analyzed using a linear mixed model. To quantify the relationship between tumor level trajectories and clinical outcomes, a joint model was used. The final linear mixed‐effects regression model included a random intercept at the patient level and a random slope for the linear follow‐up time. The final model for the follow‐up time specification (linear or quadratic) was selected based on the lowest value of Akaike's information criterion (AIC). The model parameters were estimated using maximum likelihood, and an independent variance–covariance structure was assumed for the random effects. The joint model was specified as a linear mixed growth model with random intercept at the patient level and random slope for linear follow‐up time for the longitudinal component, a Weibull model for the time‐to‐event component, and a current association specification of the association parameter α and an unstructured variance–covariance matrix. The rate of hVAF/iTF change over follow‐up time as estimated with a ‘1st derivative’ specification of α [20]. Patient‐specific outcome predictions according to their hVAF/iTF trajectories were obtained using the Stata routine stjmcsurv based on the dynamic prediction approach of Rizopoulos [20, 21]. Longitudinal data were analyzed using stata 18.5 (Stat Corp., Houston, TX, USA).

## 3 Results

### 3.1 Patients' characteristics

A total of 30 patients were enrolled at the Medical University of Vienna (Table 1), including 18 men (60%) and 12 (4%) women with a median age of 66 years (range, 41–87). In 10 patients (33%), the primary tumor was located on the right side and in 20 patients (67%) on the left side (left colon flexure or aboral) colon, respectively. Two‐thirds of the patients (67%) had an ECOG performance status of 0, while eight patients (27%) had an ECOG of 1 and two patients with an ECOG performance status scale of 2 (6%). According to RECIST 1.1 criteria, two patients had a PR (6%) and seven patients (23%) had an SD leading to a disease control rate (DCR) of 30% (9 patients). A total of 20 patients (67%) presented PD and did not respond to trifluridine/tipiralcil treatment. One patient dropped out before the first radiological evaluation was performed.

**Table 1:** Clinical characteristics of study patients.

Patients	*n* (%)
	30 (100)
Age (years)	66 (range, 41–87)
Gender	
Female	12 (40)
Male	18 (60)
Localization	
Right	10 (33)
Left	20 (67)
ECOG	
0	20 (67)
1	8 (27)
2	2 (6)
Therapy outcome	
CR	0 (0)
PR	2 (6)
SD	7 (23)
PD	20 (67)
NA	1 (4)
Overall response	2 (7)
Disease control	9 (30)
Survival	Median (months)
OS first diagnosis	63 (range, 14.7–305.3)
OS treatment	8.0 (1.1–27.8)
PFS	3.2 (1.0–16.1)
Metastases	
1	10 (33.3)
2	11 (36.7)
3	6 (20.0)
4	3 (10.0)
Prior lines of treatment	
2	13 (43.4)
3	16 (53.3)
4	1 (3.3)

The median follow‐up time in our study cohort was 16.6 months (95% CI: 11.9–24.5). The median OS since the first diagnosis was 66.4 months (range, 14.7–304.8), while the median OS since the start of targeted treatment (BL) was 8.1 months (range, 1.1–27.8) (Fig. S1). Median PFS upon FTD/TPI treatment was dismal at 3.2 months.

### 3.2 Molecular profiling results

**Fig. 1:**
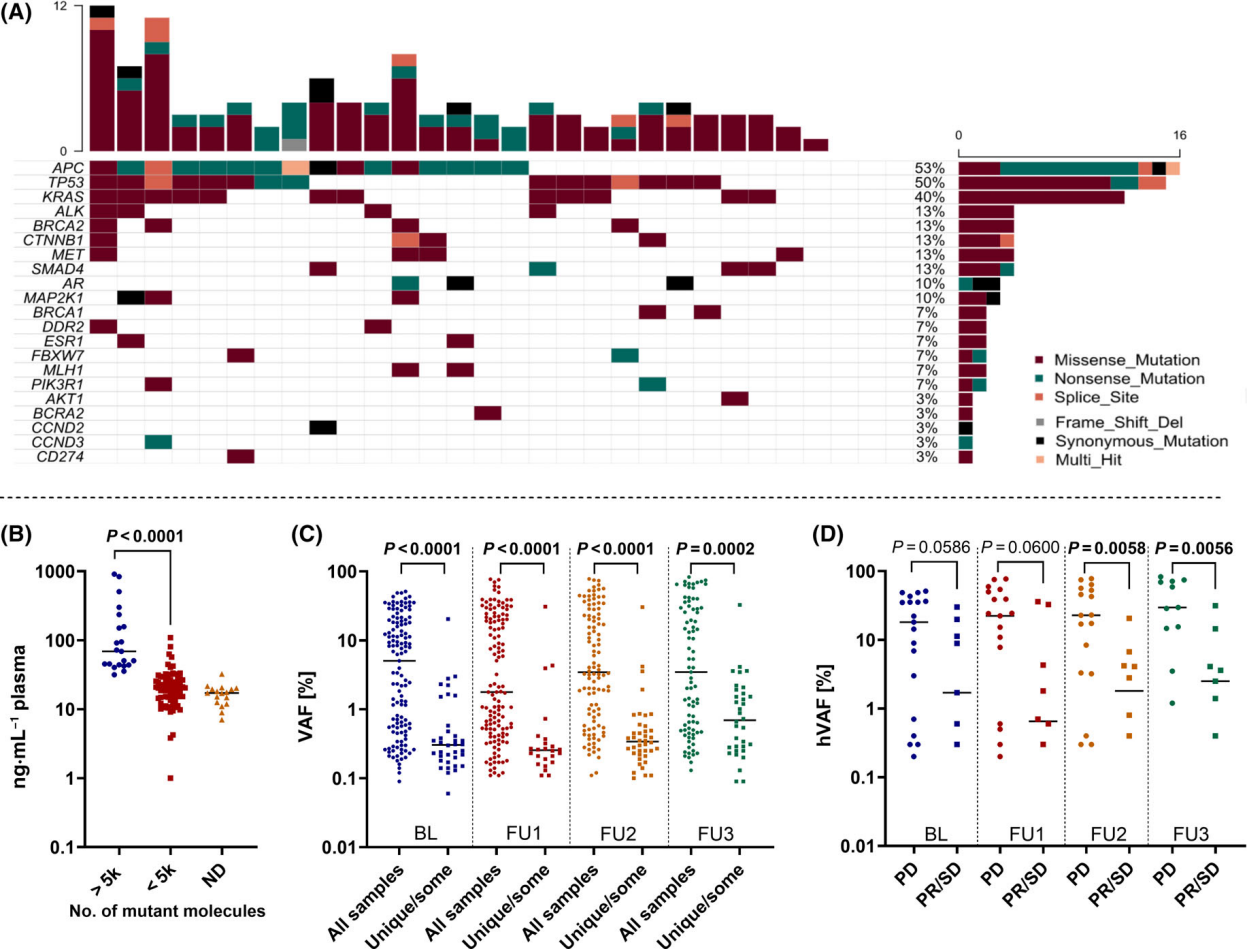
Molecular profile and distribution of circulating tumor DNA (ctDNA) levels at various time points. (A) Oncoprint of the 21 most frequently mutated genes out of a 77‐gene panel. Shown is an overview of genomic alterations (missense‐, nonsense‐, splice site‐, frameshift‐, synonymous mutations, multi hit mutations, color code see right key) in selected genes (rows) affecting individual samples (columns). Column specific histograms refer to all identified mutations. (B) Distribution of cell free DNA (cfDNA) concentration of samples with more than 5000 mutant molecules (< 5k) of the highest variant, samples with less than 5000 mutant molecules (< 5k), and samples with no ctDNA detected (ND). Pairwise comparison using Mann–Whitney test. (C) Distribution of variant allele frequency (VAF) of mutations identified in all samples (BL, baseline; FU, follow‐up) compared to VAFs of mutations that were not identified in all samples. Pairwise comparison using Mann–Whitney test. (D) Highest variant allele frequency (hVAF) at baseline (BL) and three follow‐ups (FU1‐3) stratified by best response (PD, progressive disease; PR, partial response; SD, stable disease). Line denotes the median hVAF. Pairwise comparison (PD vs. PR/SD) using Mann–Whitney test. Statistically significant *P*‐values of < 0.05 are indicated in bold.

cfDNA quantification revealed a median concentration of cfDNA of 21.6 ng·mL^−1^ plasma (range, 7.0–907.8 ng·mL^−1^ plasma). Samples with more than 5000 mutant molecules of the mutation with the hVAF had significantly higher cfDNA concentration compared to samples with less than 5000 mutant molecules (Fig. 1B and Fig. S2). Prior to treatment initiation (BL), at least one mutation could be identified in 27/30 patients (90%), with an average of 5.7 mutations (range 0–21) per patient. In three patients, no mutation could be detected at any time point, all of whom had PR or SD as the best response, consistent with a low tumor fraction (iTF, < 3%) calculated from ichorCNA.

As expected, the most frequently mutated genes included *APC*, *TP53* and *KRAS* and in the majority of cases, these CRC driver genes harbored clearly pathogenic mutations with the hVAF (Fig. 1A). Most variants were missense mutations followed by nonsense and splice mutations, while Indels were detected in only six patients. Most mutations persisted in all follow‐up samples (Fig. S3). However, new mutations occurred in some cases, or mutations were no longer detectable in FU samples (Table S4). Overall, VAFs of mutations were significantly higher when detected in both baseline and all consecutive follow‐up samples, compared to mutations not present in all samples (Fig. 1C).

### 3.3 ctDNA levels are associated with disease control

Levels of ctDNA assessed as aVAF or hVAF significantly correlated with an unbiased genomewide assessment of the tumor fraction (iTF) (Pearson correlation, aVAF/iTF *r* = 0.7415 and hVAF/iTF *r* = 0.8899) (Fig. S4). In contrast, the mutant molecules per mL plasma did not accurately reflect the iTF (Fig. S4). However, when outlier samples with extremely high numbers of mutant molecules (hMM > 5000) were removed, the correlation improved (hVAF vs. iTF, *r* = 0.7164) and the strongest correlation was observed between hMM and hVAF (*r* = 0.9083) (Fig. S5).

Since hVAF provided the best proxy for the iTF, this measure was used for further analyses. Overall, ctDNA levels at BL were high, with 14 patients (46.7%) having hVAFs of 10% or higher. ctDNA levels were not significantly different between the various time points, with median hVAFs of 8.9%, 6.1%, 6.7%, and 14.6% at BL, FU1, FU2, and FU3, respectively (Fig. S6). In contrast, when we stratified by the best response, patients with PD had significantly higher ctDNA levels at FU2 (1.8% vs. 22.8%, *P* = 0.006) and FU3 (2.5% vs. 29.6%, *P* = 0.006) compared to patients who achieved a PR or SD (Fig. 1D). A similar numerical trend, although not statistically significant, was observed at BL and FU1. Considering only variants present in all samples, patients with PD had significantly higher hVAF levels at all time points compared to patients with PR/SD (Fig. S7).

Moreover, we investigated whether ctDNA levels are associated with clinical parameters. We found weak to moderate but significant positive associations of tumor levels (hVAF) with CA19‐9 (*R* = 0.416, *P* = 0.040), thrombocytes (*R* = 0.432, *P* = 0.024), neutrophils (*R* = 0.545, *P* = 0.020), LDH (*R* = 0.587, *P* = 0.001), and CRP (*R* = 0.421, *P* = 0.026) (Table S5).

### 3.4 Elevated ctDNA levels are associated with inferior survival

**Fig. 2:**
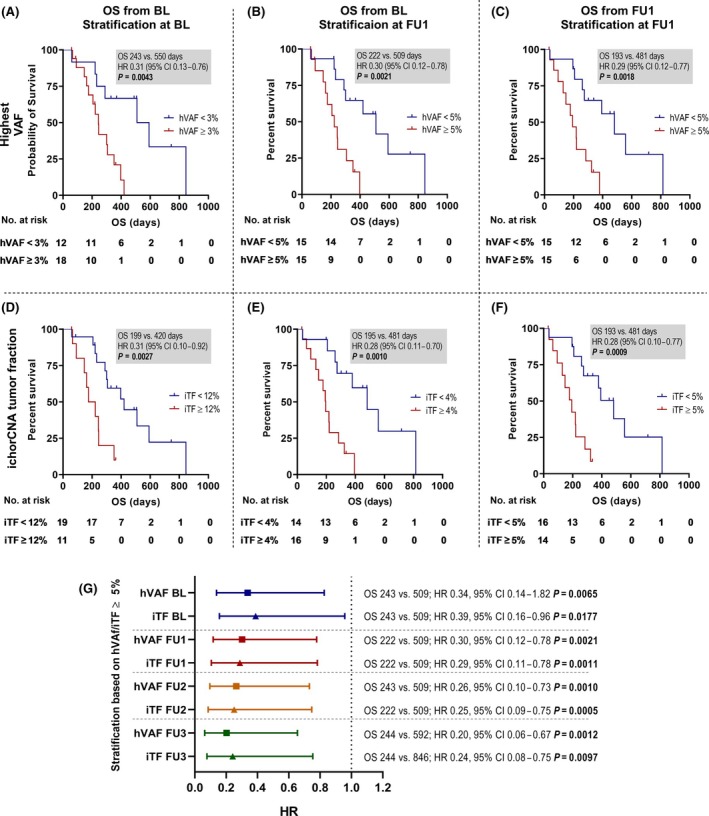
Overall survival of the cohort stratified by tumor levels. Kaplan–Meier curves of overall survival (OS) calculated from the first blood sample (A, B, D, E) or the first follow‐up (C, F) for the highest variant allele frequency (hVAF) (upper panel, A‐C) or the ichorCNA‐based tumor fraction (iTF) (lower panel, D–F) using log‐rank (Mantel‐Cox) test. Shown are the thresholds that best discriminated OS (see Fig. S8). (G) Forest plot showing the hazard ratio (HR) for OS calculated from the first blood sample at all available time points for hVAF and iTF (lower panel).

Next, we investigated whether ctDNA levels are associated with OS. To this end, we first tested several cut‐offs ranging from 1% to 15% ctDNA level at BL and FU1. At BL, all cutoffs revealed a significantly inferior survival if the ctDNA level was above the threshold (Fig. S8), with an hVAF of 3% being the most discriminative (HR: 0.31, 95% CI: 0.13–0.76 *P* = 0.0043) (Fig. 2A and Fig. S9). At FU1, hVAF > 5% revealed the best stratification with respect to OS calculated from both BL (HR 0.30, 95% CI 0.12–0.78 *P* = 0.0021) (Fig. 2B and Fig. S10) and FU1 (HR 0.29, 95% CI 0.12–0.77 *P* = 0.0018) (Fig. 2C and Fig. S11), respectively. When we used iTF to stratify patients into high and low tumor levels, the most discriminative cut‐off at BL was 12% (HR 0.31, 95% CI 0.10–0.92 *P* = 0.0027) (Fig. 2D and Fig. S12), whereas at FU1 the best separation of the curves was observed at 4% (HR 0.28 95% CI 0.11–0.70 *P* = 0.0010) (Fig. 2E and Fig. S13). When considering OS from the second blood draw (FU1), patients with iTF levels above 5% had significantly worse survival with a median OS of 6.3 months compared to patients with lower levels, who had a median OS of 15.8 months (HR 0.28, 95% CI 0.10–0.77 *P* = 0.0009) (Fig. 2F and Fig. S14). Since time‐point‐ and methodology‐related or cohort‐dependent cut‐offs are not applicable for widespread clinical use, we tested a 5% cut‐off for both proxies hVAF and iTF at all time points. Regardless of the timing of the blood sampling, the 5% threshold was always significantly associated with OS (Fig. 2G).

In a univariate Cox regression analysis including clinical variables, iTF at FU1 and LDH (either dichotomized or as continuous variables) were found to be significantly associated with OS (Table S6). Due to the relatively small number of patients, multivariate analysis was limited in our cohort. Nevertheless, since performance status and the number of metastases were previously identified as predictors for OS, we included these variables in a multivariate model, in which both iTF at FU1 using a cut‐off of 5% and ECOG remained highly significant for OS (HR: 4.90, 95% CI: 1.496–17.71) (Tables S7 and S8). The poor outcome measures associated with the ctDNA levels also remained significant when elevated LDH was added to the model (Tables S7 and S8).

### 3.5 ctDNA trajectories predict overall survival

**Fig. 3:**
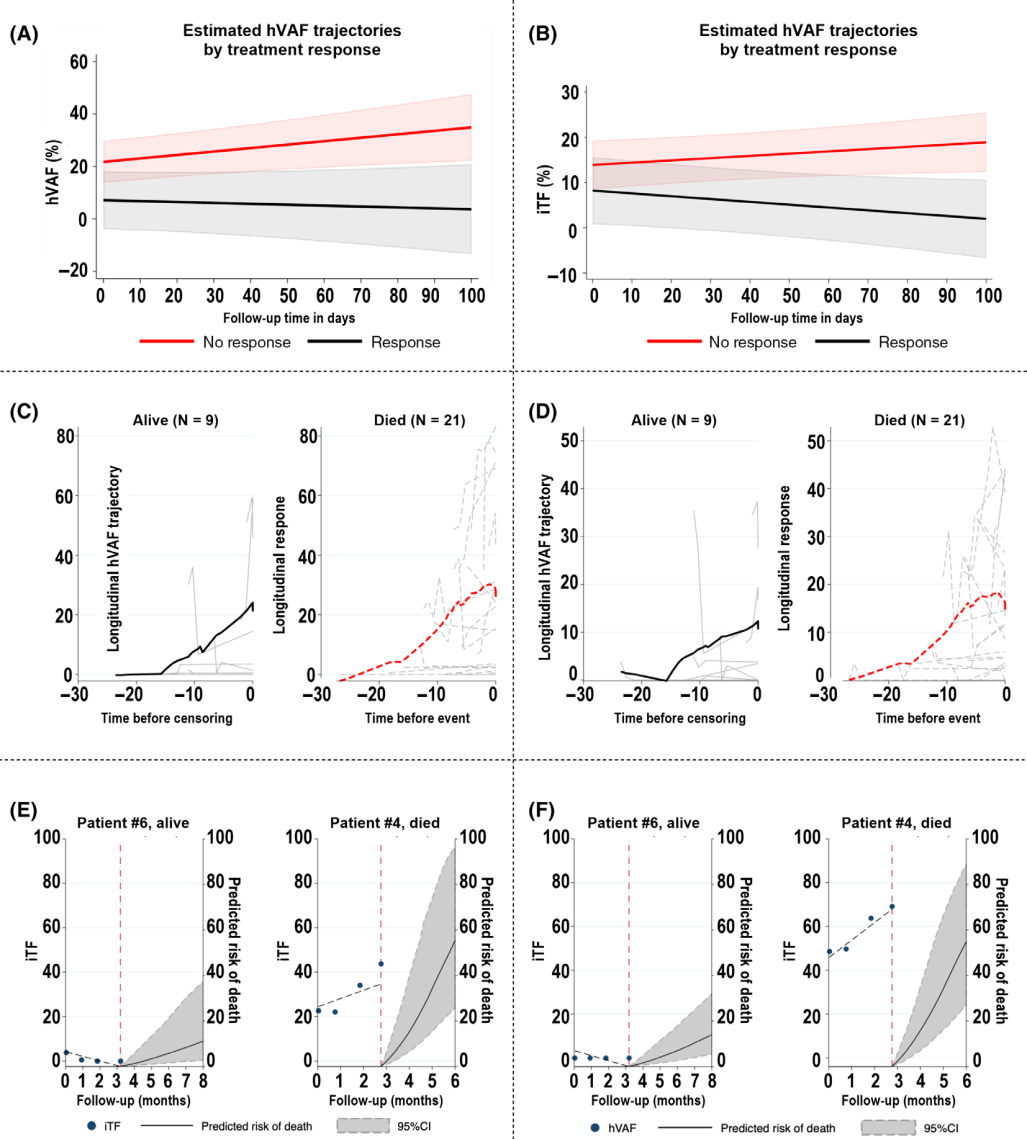
Prognostic evaluation of circulating tumor DNA trajectories. Evolution of (A) highest variant allele frequency (hVAF) and (B) ichorCNA‐based tumor fraction (iTF) trajectories over follow‐up time in patients with and without treatment response. Data are from a linear mixed‐effects regression model with a random intercept at the patient level and a random slope for linear follow‐up time (DCR, disease control rate; hVAF, highest variant allele frequency). Line plot of (C) hVAF and (D) iTF trajectories in patients who remained alive (*N* = 9, left panel, black bold solid line) and who died (*N* = 21, right panel, red bold dashed line) during follow‐up time. Each gray line represents the hVAF trajectory of a single patient. The bold solid lines represent moving averages (locally weighted sum‐of‐squares (LOWESS) nonparametric smoother). The time on the *x*‐axis of both panels is inverted, i.e., it represents the time before death or censoring without death. (E, F) Personalized 6‐month mortality risk prediction (solid black line with 95% confidence interval in gray) for two patients according to their actual individual hVAF/iTF trajectories (dotted gray line). Red dashed‐dotted line indicates the last study visit. Predictions were obtained from the joint model with the current value association structure.

As specific cut‐offs of ctDNA levels predicting response may not be reproducible in other cohorts, we investigated the longitudinal evolution of hVAF levels under treatment. To this end, we applied a mixed model with linear growth of hVAF levels, a random intercept at the patient level, and a random slope for linear follow‐up time. For this analysis, we included 29 patients with available response data along with 105 hVAF measurements (average hVAF measurements per patient: 3.6, range 2–4). According to this model, patients with treatment response had significantly lower hVAF levels at any follow‐up time compared to patients without treatment response (estimated difference = −13.8, 95% CI: (−27.29) to (−0.21), *P* = 0.047). hVAF levels significantly changed over time (change = 0.13, 95% CI: 0.04–0.23, *P* = 0.007) and hVAF trajectories were significantly higher in patients without treatment response compared to those with treatment response (interaction *P*‐value for linear follow‐up time = 0.037) (Fig. 3A). We applied the same model for iTF levels, and also observed significant interactions for the linear follow‐up time (*P* = 0.020), indicating that iTF trajectories over time differed significantly between patients with and without treatment response. However, the estimated difference in iTF levels at any follow‐up time between patients with and without treatment response (*P* = 0.225) and the change in iTF levels over time (*P* = 0.092) were not statistically significant (Fig. 3B).

Next, in univariable joint modeling of hVAF trajectories and time‐to‐death (Fig. 3C), an elevated hVAF trajectory over time was associated with a higher risk of death (HR of 1 unit increase of hVAF at any time of follow‐up of 1.03, 95% CI: 1.01–1.04, *P* < 0.001). Likewise, an elevated iTF trajectory over time was associated with a higher risk of death (HR of 1 unit increase of iTF at any time of follow‐up: 1.06, 95% CI: 1.02–1.10, *P* = 0.003) (Fig. 3D). Furthermore, a higher rate of hVAF increase over time was also strongly prognostic for an increased risk of death (HR: 1.27, 95% CI: 1.06–1.52, *P* = 0.010). The same trend was observed for an iTF increase over time, although this result was not statistically significant (HR: 1.20, 95% CI: 0.91–5.56, *P* = 0.081). Finally, with this joint model, personalized risk predictions of mortality conditional on individual patients' hVAF trajectories could be obtained. For example, patient #6 had constantly low hVAF levels over the follow‐up time. His predicted mortality risk 6 months after his last study visit was below 20%, and indeed, this patient was still alive after 24 months of follow‐up time (Fig. 3E). Patient #4 had a high hVAF baseline level, followed by a significant increase during the follow‐up time. This patient died after 8 months of follow‐up time, which was consistent with the predicted 6‐month mortality risk of 85%. iTF trajectories yielded very similar results to those obtained with hVAF measurements (Fig. 3F).

## 4 Discussion

Recent introductions of novel therapeutic agents in the late‐line setting have significantly improved the prognosis of mCRC. While targeted therapies, which specifically block essential biochemical pathways or mutant proteins, require knowledge about the molecular profile of the tumor, the antimetabolite conjugate FTD/TPI is active in all molecular subgroups of mCRC. FTD/TPI is independent of pretreatments with biological agents such as anti‐VEGF and/or anti‐EGFR antibodies or regorafenib, well tolerated and can improve not only OS but also delay the deterioration of the performance status [22]. However, in this heavily pretreated patient population, disease control by FTD/TPI alone – as for other effective drugs in this setting – is achieved in less than half of the patients and a subset of patients experience severe adverse events compromising quality of life. Thus, biomarkers predicting a benefit from this late‐line treatment are urged.

We investigated whether ctDNA can predict response to FTD/TPI and/or long‐term benefit in heavily pretreated mCRC. To this end, we assessed the tumor fraction prior to treatment start and during the first 12 weeks of treatment and correlated ctDNA levels and trajectories with response and OS. The median OS in our study was 8.1 months, which was superior in a cross‐over comparison with the pivotal clinical phase III trial RECOURSE [6]. However, with only two patients achieving PR and nine patients having SD as best response according to RECIST 1.1 criteria, the disease control rate (30%) was lower than previously reported [6], but this might be due to the limited number of patients studied. It is noteworthy that in this heavily pretreated patient population, strong responses to systemic treatment are not expected. The treatment aim in this setting is defined as improving the prognosis and maintaining the performance status of the patients by achieving control of the disease. Therefore, a well‐powered analysis of ctDNA and treatment response was impossible.

Using a 77‐gene panel, we were able to identify ctDNA in 90% of our patients, and in line with the advanced stages, the majority of patients had elevated tumor fractions at BL and during the first 12 weeks of treatment. When using hVAF as a proxy for the tumor levels, 17 (23.3%) and 14 (46.6%) patients had tumor fractions of 5% and 10% or higher at BL, respectively. 30% of the patients even had ctDNA levels above 25%, confirming CRC as a tumor entity that sheds high amounts of DNA into the circulation [23]. The fact that ctDNA levels in our cohort were exceptionally high was not unexpected since almost all patients had liver metastases that were recently shown to exhibit the highest degree of ctDNA shedding [23]. Nevertheless, patients with lower levels were more likely to achieve disease control.

More importantly, high ctDNA levels were associated with worse survival, which aligns with previous reports [12, 14, 24, 25]. In contrast to one of our previous studies [14], here we compared a mutation‐based assessment of tumor fraction (hVAF) with the aneuploidy‐based estimate ichorCNA (iTF) as proxies for ctDNA levels. The hVAF is most likely the most accurate representation of the actual tumor fraction since it can be assumed that it originates from truncal mutations that are represented in the majority of tumor cells. In fact, in most cases, the highest variant was clearly pathogenic and located in one of the key initiators of colorectal carcinogenesis, such as *APC* (26.6%), *TP53* (26.6%) and *KRAS* (10%). At the same time, driver mutations may be overestimated when located in regions affected by copy number alterations [26]. In contrast, the average of all mutations is very prone to subclonal variations and might underestimate the actual tumor fraction. Moreover, when corresponding white blood cells are not included in the assay, variants originating from clonal hematopoiesis might further affect the quantification [27]. Estimates of tumor fraction based on untargeted approaches, such as sWGS, maybe more unbiased but come with limited sensitivities and have a lower dynamic range. However, in an advanced setting such as the presented study, where the majority of patients present elevated ctDNA levels, the most discriminative prognostic cut‐offs of tumor levels were in a range that could be reliably assessed using sWGS. The best stratification was achieved after 4 weeks of treatment using an iTF of 5% as a cut‐off. This time point is clinically useful since clinicians may decide whether therapy should be continued or discontinued. A major advantage of sWGS over high‐resolution gene panels is the fast turn‐around time and the low costs, making it a beneficial approach for continuous and tight monitoring strategies. Moreover, this approach is not confounded by clonal hematopoiesis.

On the other hand, besides a higher sensitivity, mutation‐based analyses provide a more comprehensive picture of the molecular landscape of the tumor, and potentially druggable targets might additionally be identified. In our cohort, we did not observe any clonal switches or prominent new changes within 3 months of treatment. However, in some patients, several low‐level mutations were detected only in some samples that either disappeared or newly occurred in follow‐up samples. It remains unclear whether these inconsistencies are a consequence of sampling biases or subclonality. Most variants that were identified in all samples were located in truncal tumor suppressors and oncogenes commonly mutated in CRC, such as *APC*, *KRAS* and *TP53*.

Taken together, the most useful monitoring strategy may include a combination of both approaches, a continuous, quantitative assessment of tumor levels using sWGS with the addition of a gene panel at the time of progression to screen to actionable targets. Based on our data we propose a 5% cut‐off for prognostication irrespective of the methods or the evaluated time point. However, for clinical applicability this need to be reproduced and validated in larger cohorts.

Since ctDNA trajectories harbor additional important dynamic information on the development of disease progression, we additionally assessed longitudinal associations of ctDNA and survival. Previously, we have demonstrated that joint models can assess the relationship of quantitative changes of ctDNA assessed with mFAST‐SeqS – an amplicon‐based approach that quantifies the degree of aneuploidy in a sample and acts as a surrogate for ctDNA levels – with the risk of developing progressive disease and/or death in breast cancer [28]. In our CRC cohort, we could confirm the prognostic value of such models using both hVAF and iTF. Our data revealed that ctDNA trajectories were significantly associated with a higher risk of progression and death. In contrast, patients with low ctDNA trajectories had significantly better prognoses. Therefore, such models allow predictions of mortality conditional on individual patients' tumor level trajectories. Particularly in CRC, cytotoxic therapies play an important role in treating late‐stage disease for symptom palliation and prolonging survival. However, administration of chemotherapy in late‐stage patients could cause unnecessary suffering to patients and cost to society, and there is evidence that the use of chemotherapy near the end of life is not related to its likelihood of providing benefit [29]. Moreover, antitumor therapy may be toxic and potentially life‐threatening for some patients, resulting in a poorer quality of life. Therefore, de‐escalation of systemic treatment is currently the subject of numerous analyses [30]. From a clinical standpoint, our model may enable identification of patients at high risk of death in a short time that could be spared from aggressive therapy with the prospect of appropriate quality of life in their remaining lifetime. On the other hand, patients with a good prognosis may truly benefit from further systemic (or targeted) treatment to prolong the period to the exacerbation of the disease and, thereby, OS. Of course, this needs to be evaluated in well‐powered, large cohorts.

The models used in this study can be continuously adjusted (i.e., if more time points are available) to estimate the future risk of disease progression and can be applied to different types of datasets and ctDNA proxies. In general, ctDNA levels can be assessed in many ways.

## 5 Conclusions

In summary, in this study, we demonstrated for the first time the use of applied joint/mixed models to quantify the relationship between ctDNA trajectories and clinical outcomes in advanced CRC patients. Despite the limitations of a small sample size and the fact that not all time points were available in every patient, which impairs the generalization of our conclusions, we demonstrate that ctDNA can identify predictive as well as prognostic signals to identify patients who might have a long‐term benefit from FTD/TPI in late‐line treatment of mCRC. As the combination of bevacizumab with FTD/TPI will likely define a new standard in pretreated mCRC patients, the data presented here might be hypothesis generating to identify those patients who might have a long‐term benefit from this third‐line treatment.

## Conflict of interest

All authors except GWP and EH declare no conflict of interest. GWP received honoraria for advisory boards and speaker's fee from Bayer, Servier, Roche, Merck, Amgen, Sanofi, Lilly, Pierre‐Fabre, AstraZeneca, Daiichi, BMS and MSD. EH has received unrelated funding from Illumina, Roche, Servier, Freenome, and PreAnalytiX, and received honoraria from Roche and Astra Zeneca for advisory boards unrelated to our study.

## Author contributions

MU and GWP initiated the study, contributed patient material and provided clinical record. RG, MB and CB performed laboratory work including sample management and sequencing. EH, ND, and SK performed analysis of sequencing data and statistics. EH and GWP drafted the manuscript. All authors read and approved the final manuscript.

## Supporting information


**Fig. S1.** Survival of the cohort.
**Fig. S2.** Distribution of cfDNA concentration (ng·mL^−1^ plasma) based on the identified number of mutant molecules (hMM).
**Fig. S3.** Number of mutations identified across time points.
**Fig. S4.** Correlation of ctDNA levels assessed with various measures.
**Fig. S5.** Correlation of ctDNA levels assessed with various measures.
**Fig. S6.** Distribution of cfDNA concentration and ctDNA levels.
**Fig. S7.** ctDNA levels stratified by best response.
**Fig. S8.** Hazard ratios for all various ctDNA level thresholds.
**Fig. S9.** Kaplan–Meier curves of OS calculated from the first blood sample.
**Fig. S10.** Kaplan–Meier curves of OS calculated from the first blood sample.
**Fig. S11.** Kaplan–Meier curves of OS calculated from the first follow‐up sample.
**Fig. S12.** Kaplan–Meier curves of OS calculated from the first blood sample.
**Fig. S13.** Kaplan–Meier curves of OS calculated from the first blood sample.
**Fig. S14.** Kaplan–Meier curves of OS calculated from the first follow‐up sample.
**Table S1.** Gene list and types of detectable alterations for the AVENIO Expanded Panel.
**Table S2.** The sequencing coverage and quality statistics of the enriched data.
**Table S3.** The sequencing coverage and quality statistics of the shallow whole genome sequencing.
**Table S4.** Overview of detected mutations and various measures of tumor fractions.
**Table S5.** Correlation of tumor levels as hVAF (highest variant allele frequency) and blood makers.
**Table S6.** Univariate Cox regression model for OS based on ctDNA level (iTF) at FU1 and clinical variables.
**Table S7.** Multivariate Cox regression model for OS, including ctDNA levels (iTF at FU1) and clinical variables.
**Table S8.** Multivariate Cox regression model for OS, including ctDNA levels (iTF at FU1) and clinical variables.

## Data Availability

The data supporting these studies' findings are available from the corresponding authors upon request. All sequencing raw data have been deposited at the European Genome‐phenome Archive (EGA; http://www.ebi.ac.uk/ega/), hosted by the EBI, under the accession numbers EGAS00001006883.
