# Supplementary material for: Circulating tumor DNA (ctDNA) trajectories predict survival in trifluridine/tipiracil‐treated metastatic colorectal cancer patients
Source: Mol Oncol. 2025 Jan 22;19(7):2120–32. doi: 10.1002/1878-0261.13755 (PMC12234374; doi:10.1002/1878-0261.13755)
Supplement: Supplementary file 1 — Fig. S1. Survival of the cohort. Fig. S2. Distribution of cfDNA concentration (ng·mL−1 plasma) based on the identified number of mutant molecules (hMM). Fig. S3. Number of mutations identified across time points. Fig. S4. Correlation of ctDNA levels assessed with various measures. Fig. S5. Correlation of ctDNA levels assessed with various measures. Fig. S6. Distribution of cfDNA concentration and ctDNA levels. Fig. S7. ctDNA levels stratified by best response. Fig. S8. Hazard ratios for all various ctDNA level thresholds. Fig. S9. Kaplan–Meier curves of OS calculated from the first blood sample. Fig. S10. Kaplan–Meier curves of OS calculated from the first blood sample. Fig. S11. Kaplan–Meier curves of OS calculated from the first follow‐up sample. Fig. S12. Kaplan–Meier curves of OS calculated from the first blood sample. Fig. S13. Kaplan–Meier curves of OS calculated from the first blood sample. Fig. S14. Kaplan–Meier curves of OS calculated from the first follow‐up sample. Table S1. Gene list and types of detectable alterations for the AVENIO Expanded Panel. Table S2. The sequencing coverage and quality statistics of the enriched data. Table S3. The sequencing coverage and quality statistics of the shallow whole genome sequencing. Table S4. Overview of detected mutations and various measures of tumor fractions. Table S5. Correlation of tumor levels as hVAF (highest variant allele frequency) and blood makers. Table S6. Univariate Cox regression model for OS based on ctDNA level (iTF) at FU1 and clinical variables. Table S7. Multivariate Cox regression model for OS, including ctDNA levels (iTF at FU1) and clinical variables. Table S8. Multivariate Cox regression model for OS, including ctDNA levels (iTF at FU1) and clinical variables. [file MOL2-19-2120-s001.zip › Unseld et al_ctDNA_FTD_TPIl_data supplement_revised_final.docx]

**ctDNA as a predictor for survival in trifluridine/tipiracil-treated metastatic colorectal cancer patients**

**Matthias Unseld^1#^, Stefan Kühberger^2,3#^, Ricarda Graf^2^, Christine Beichler^2^, Markus Braun^2^, Nadia Dandachi^4,5^, Ellen Heitzer^2,3^*, Gerald W Prager^6^***

^1^Medical University of Vienna, Department of Medicine I, Division of Palliative Care, Waehringer Guertel 18-20, 1090 Vienna, Austria; ^2^Institute of Human Genetics, Diagnostic and Research Center for Molecular BioMedicine, Medical University of Graz, Neue Stftingtalstrasse 6, 8010 Graz, Austria; ^3^Christian Doppler Laboratory for Liquid Biopsies for Early Detection of Cancer, Medical University of Graz, Neue Stftingtalstrasse 6, 8010 Graz, Austria; ^4^Division of Oncology, Department of Internal Medicine, Medical University of Graz, Auenburggerplatz 15, 8036 Graz, Austria, ^5^Research Unit for Epigenetic and Genetic Cancer Biomarkers, Medical University of Graz, 8036 Graz, Austria, ^6^Medical University of Vienna, Department of Medicine I, Division of Oncology, Waehringer Guertel 18-20, 1090 Vienna, Austria

**#, * Contributed equally**

**Table of contents**

[Figure S1. Survival of the cohort. 2](#_Toc171679433)

[Figure S2. Distribution of cfDNA concentration [ng/ml plasma] based on the identified number of mutant molecules (hMM). 3](#_Toc171679434)

[Figure S3. Number of mutations identified across time points. 4](#_Toc171679435)

[Figure S4. Correlation of ctDNA levels assessed with various measures. 5](#_Toc171679436)

[Figure S5. Correlation of ctDNA levels assessed with various measures. 6](#_Toc171679437)

[Figure S6. Distribution of cfDNA concentration and ctDNA levels. 7](#_Toc171679438)

[Figure S7. ctDNA levels stratified by best response. 8](#_Toc171679439)

[Figure S8. Hazard ratios for all various ctDNA level thresholds. 9](#_Toc171679440)

[Figure S9. Kaplan-Meier curves of OS calculated from the first blood sample. 10](#_Toc171679441)

[Figure S10. Kaplan-Meier curves of OS calculated from the first blood sample. 11](#_Toc171679442)

[Figure S11. Kaplan-Meier curves of OS calculated from the first follow-up sample. 12](#_Toc171679443)

[Figure S12. Kaplan-Meier curves of OS calculated from the first blood sample. 13](#_Toc171679444)

[Figure S13. Kaplan-Meier curves of OS calculated from the first blood sample. 14](#_Toc171679445)

[Figure S14. Kaplan-Meier curves of OS calculated from the first follow-up sample. 15](#_Toc171679446)

[Table S1. Gene list and types of detectable alterations for the AVENIO Expanded Panel 16](#_Toc171679447)

[Table S2. The sequencing coverage and quality statistics of the enriched data 17](#_Toc171679448)

[Table S3. The sequencing coverage and quality statistics of the shallow whole genome sequencing 20](#_Toc171679449)

[Table S4. Overview of detected mutations and various measures of tumor fractions 22](#_Toc171679450)

[Table S5. Correlation of tumor levels as hVAF (highest variant allele frequency) and blood makers 23](#_Toc171679451)

[Table S6. Univariate Cox regression model for OS based on ctDNA level (iTF) at FU1 and clinical variables 24](#_Toc171679452)

[Table S7. Multivariate Cox regression model for OS, including ctDNA levels (iTF at FU1) and clinical variables 25](#_Toc171679453)

[Table S8. Multivariate Cox regression model for OS, including ctDNA levels (iTF at FU1) and clinical variables 26](#_Toc171679454)


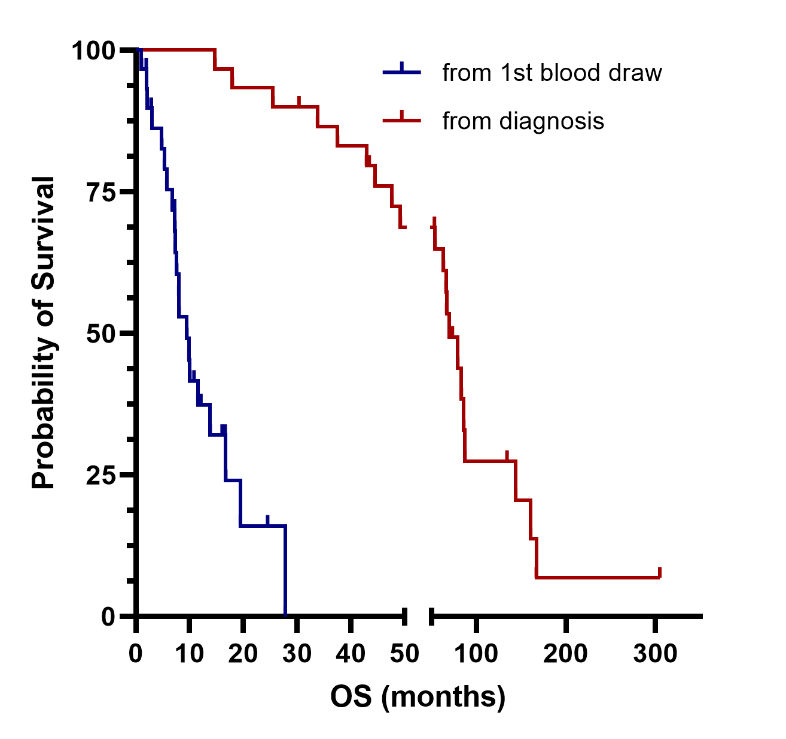


Figure S1. Survival of the cohort. Kaplan-Meier curves of **(A)** OS calculated from the first blood sample (B) Survival from the time of diagnosis


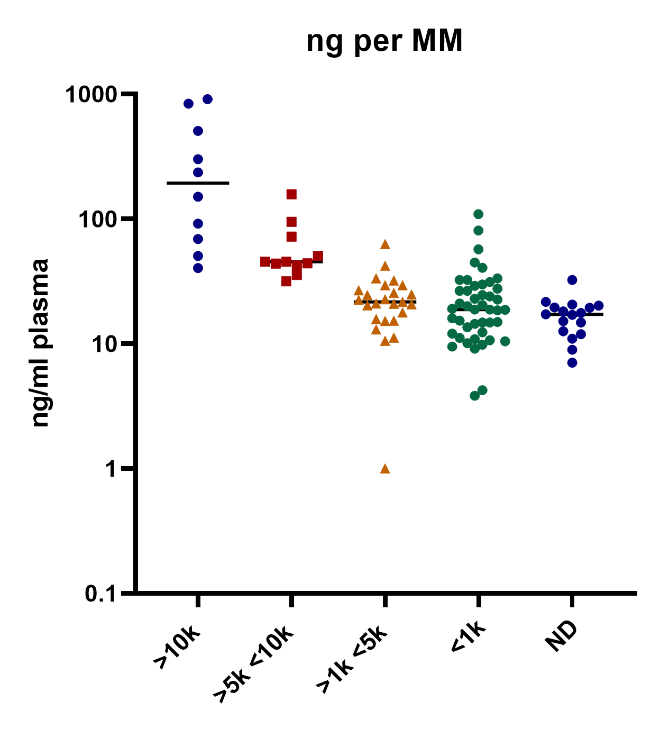


***Figure S2. Distribution of cfDNA concentration [ng/ml plasma] based on the identified number of mutant molecules (hMM).***


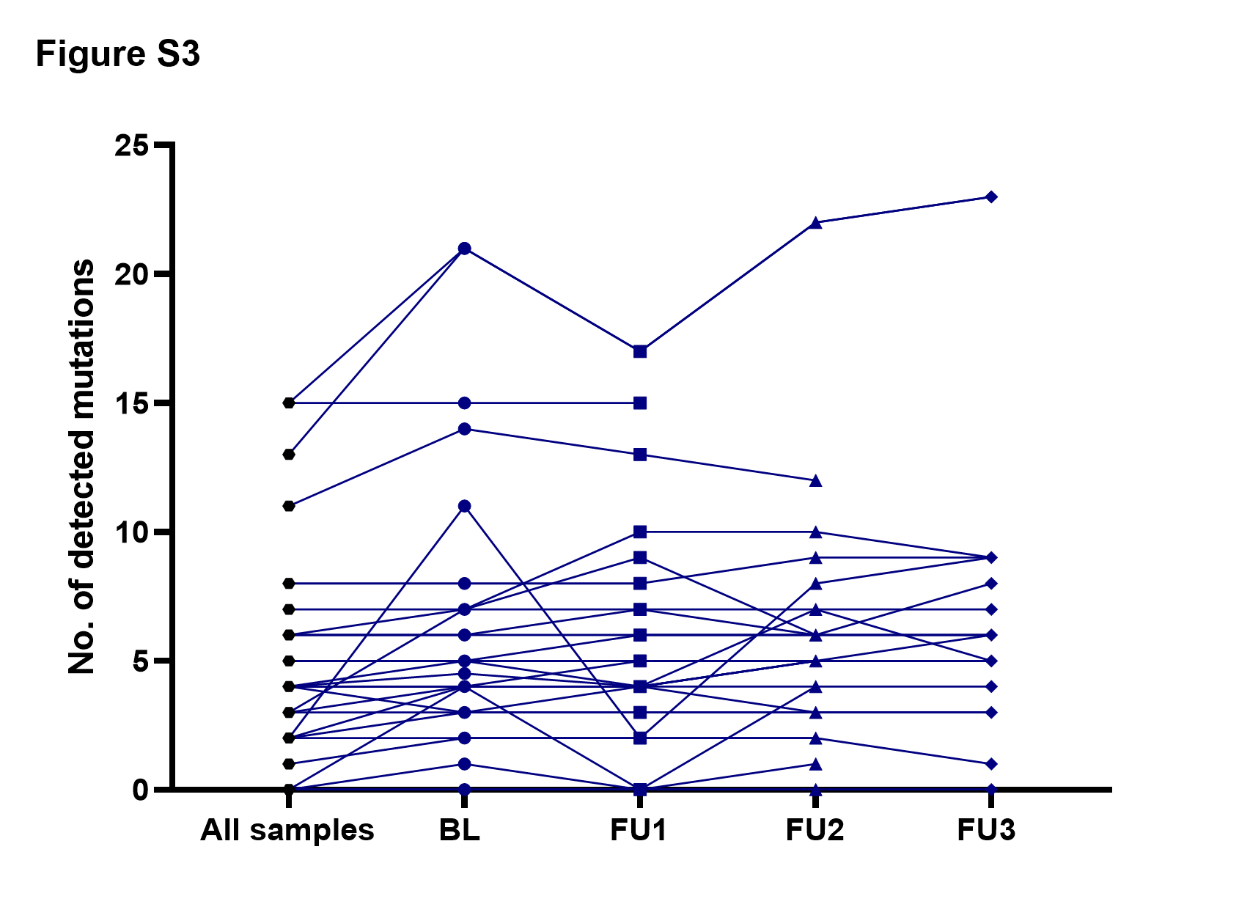


Figure S3. Number of mutations identified across time points. BL, baseline; FU1, FU2, FU3, follow-up samples collected during therapy

**
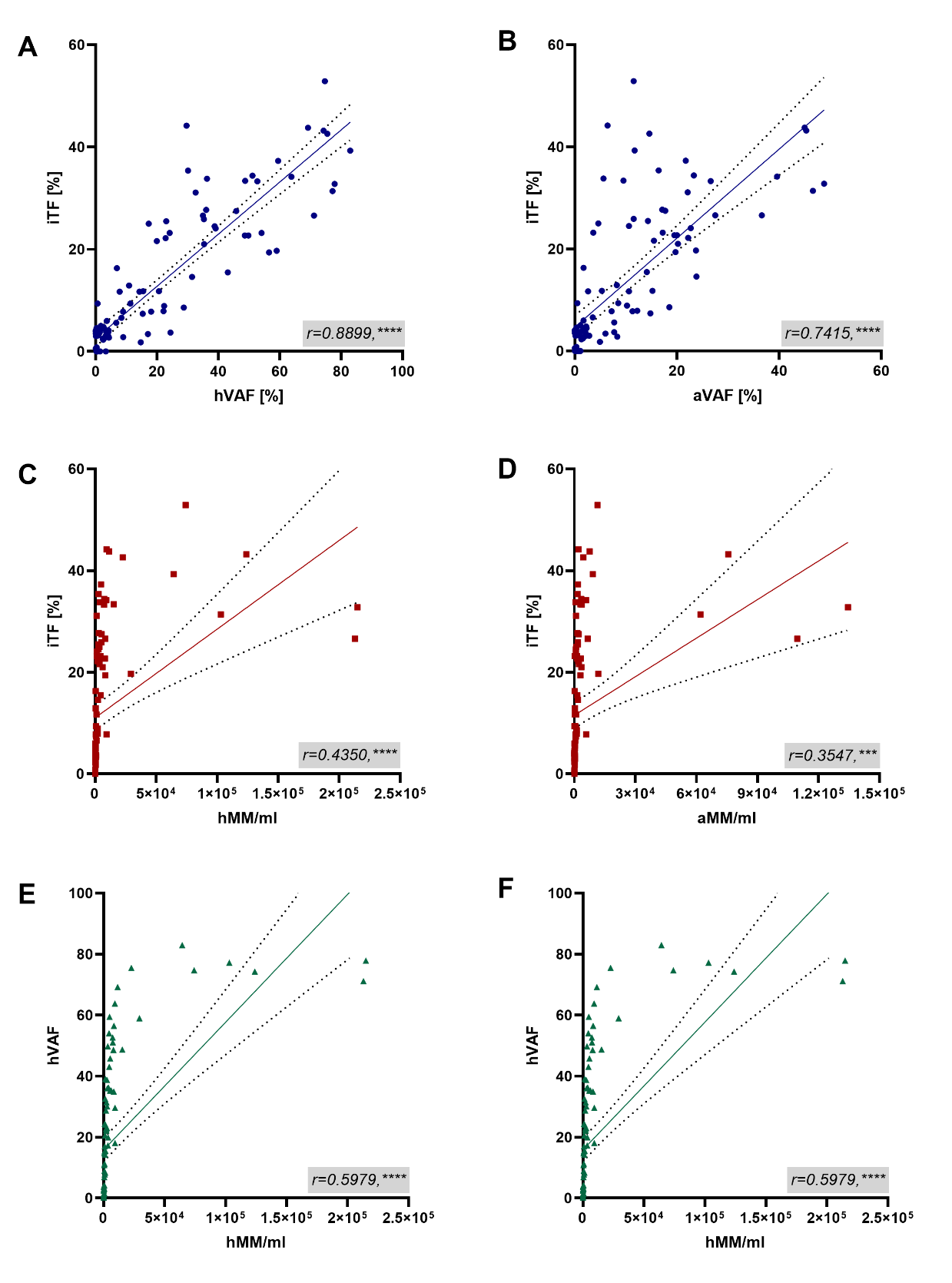
**

Figure S4. Correlation of ctDNA levels assessed with various measures. **(A-D)** Linear regressions of ctDNA levels assessed as the highest variant allele frequencies (hVAF), the average VAF of all detected mutations per patient (aVAF), the highest number of mutant molecules per ml plasma (hMM) and the average number of mutant molecules of all mutations per patient (aMM) with an untargeted measure estimated from ichorCNA (iTF). **(E-F)** Linear regressions of VAF and MM. r, Pearson correlation coefficient, **** *P<*0.001.


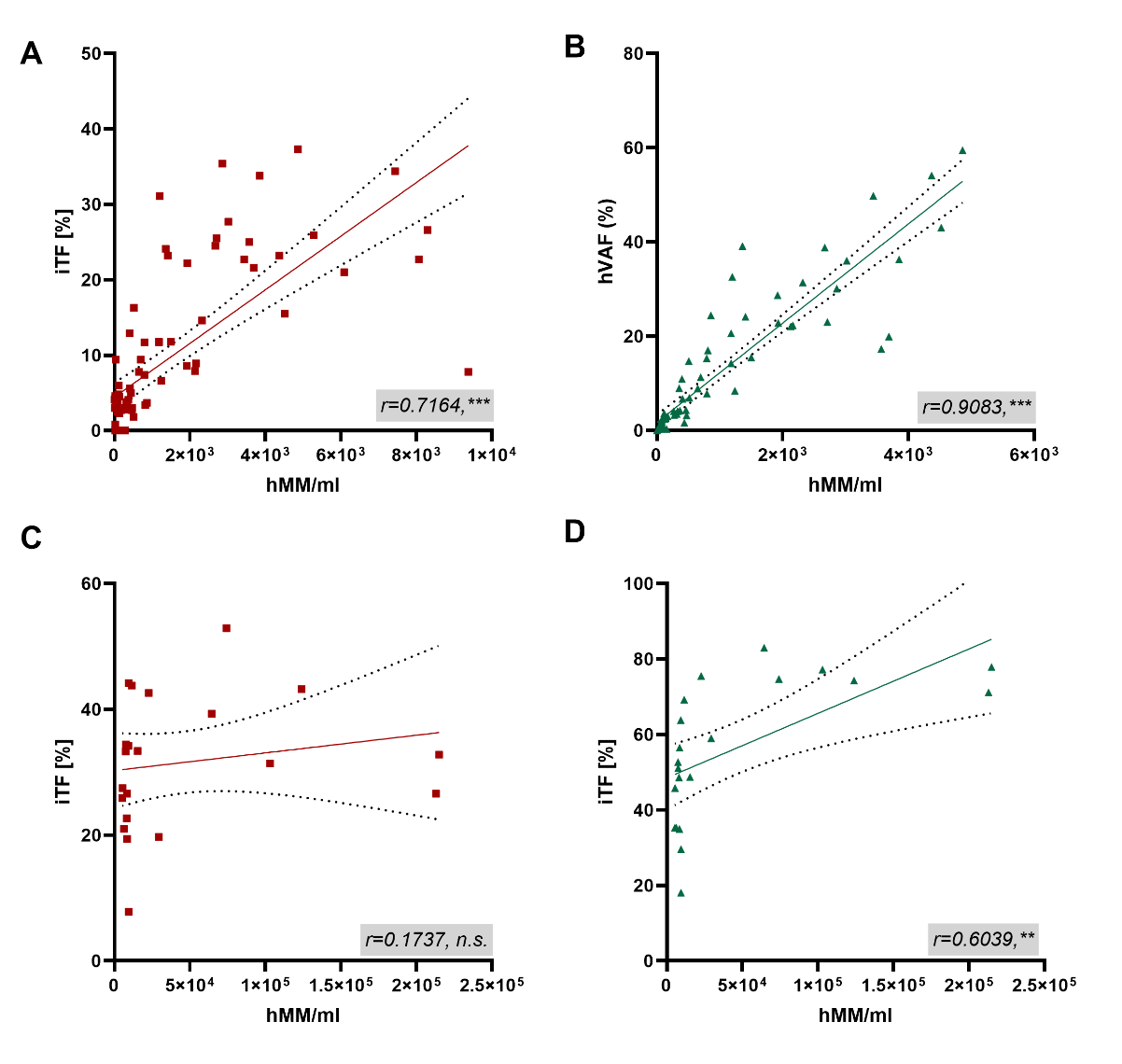


Figure S5. Correlation of ctDNA levels assessed with various measures. Linear regressions of ctDNA levels assessed as the highest number of mutant molecules per ml plasma, restricted to samples with less than 5000, with **(A)** an untargeted measure estimated from ichorCNA (iTF) and **(B)** the highest variant allele frequencies (hVAF). Linear regressions of ctDNA levels assessed as the highest number of mutant molecules per ml plasma, restricted to samples with more than 5000, with **(C)** iTF and **(B)** hVAF.


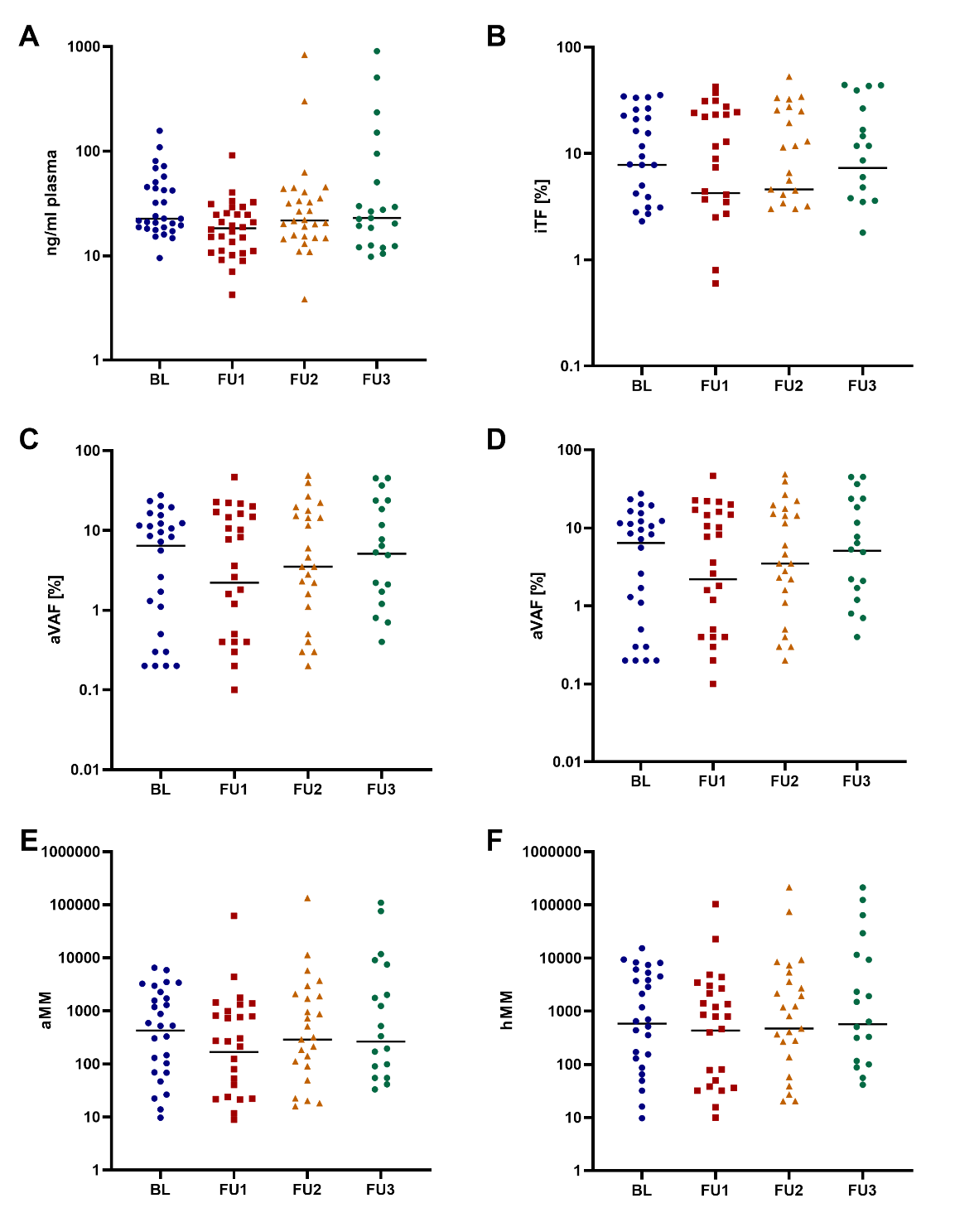


Figure S6. Distribution of cfDNA concentration and ctDNA levels. **(A)** Shown are cfDNA levels for baseline (BL) samples and three follow-up (FU) samples. Distribution of ctDNA levels assessed as **(B)** ichorCNA tumor fractions (iTF) **(C)** the average VAF of all detected mutations per patient (aVAF) **(D)** the highest variant allele frequencies (hVAF) **(E)** the average number of mutant molecules of all mutations per patient (aMM) (F) the highest number of mutant molecules per ml plasma (hMM).


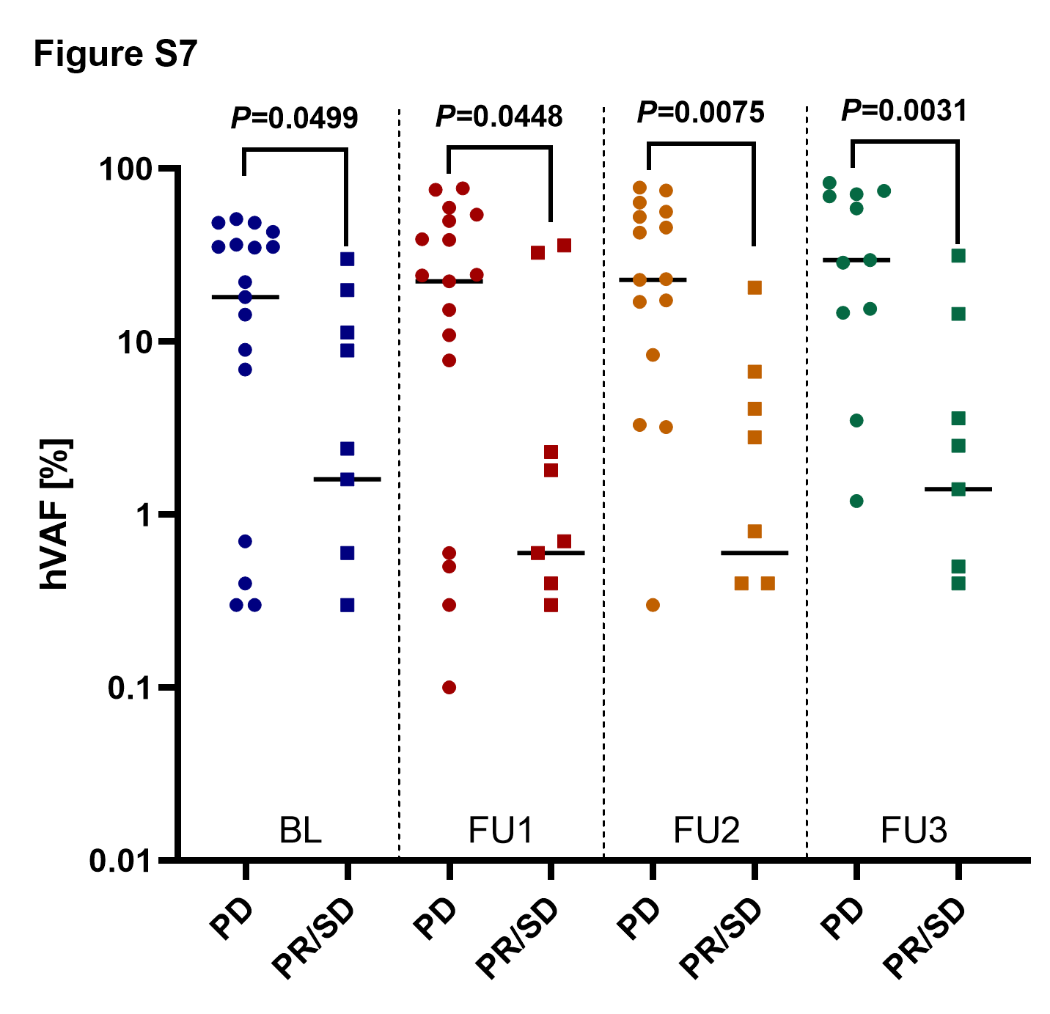


Figure S7. ctDNA levels stratified by best response. hVAF of mutation identified in all four samples stratified by best response (PD, progressive disease; PR, partial response; SD, stable disease). Pairwise comparison (PD versus PR/SD) using Mann-Whitney test.

**
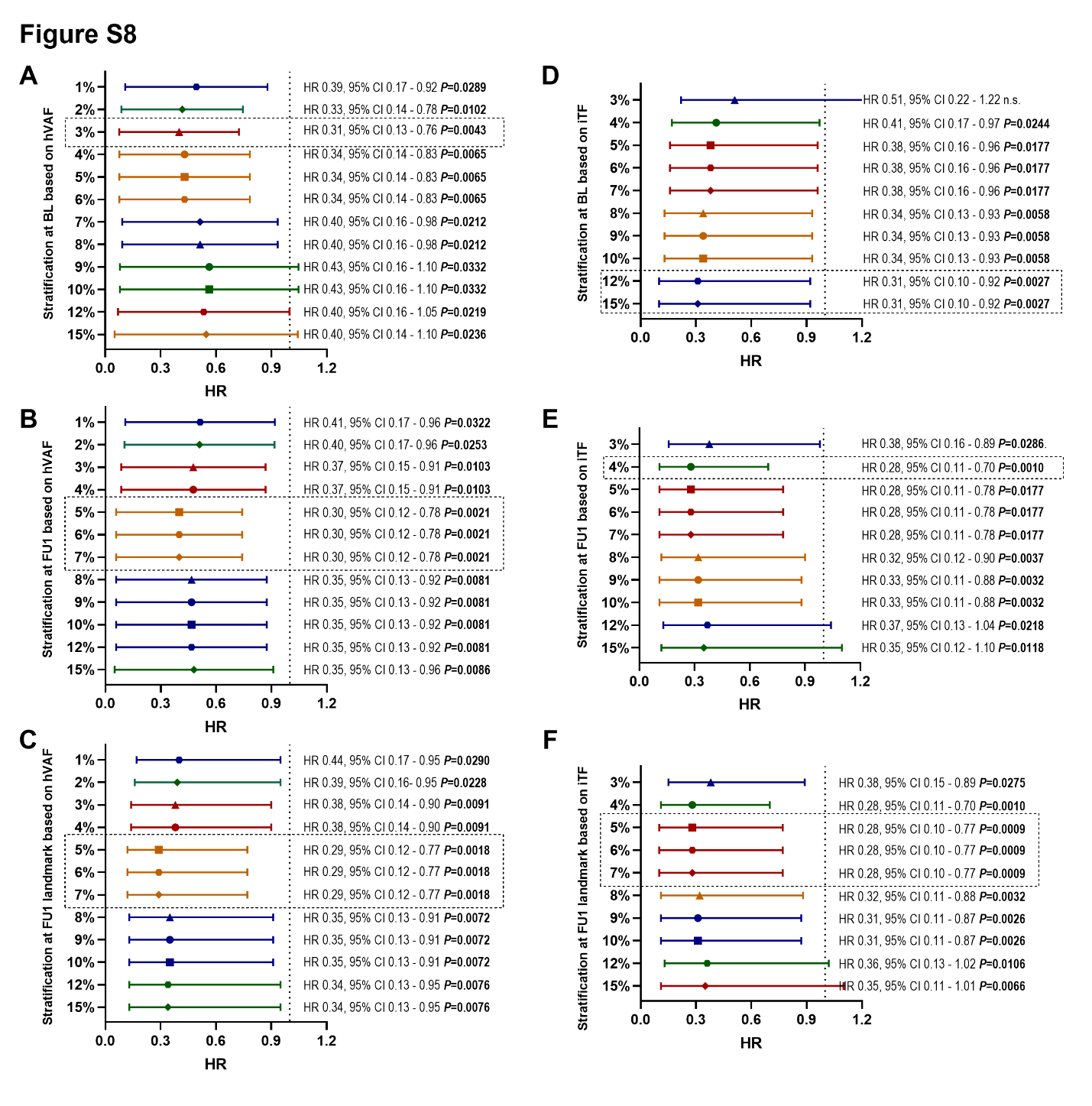
**

Figure S8. Hazard ratios for all various ctDNA level thresholds. Forest plot showing the hazard ratio (HR) and 95% confidence intervals (CI) associated with various ctDNA levels assessed as **(A-C)** the highest variant allele frequency (hVAF) or **(D-F)** ichorCNA tumor fraction (iTF) at baseline (BL) and the first follow-up (FU1). OS was calculated from BL **(A, B, D, E)** or FU1 **(C, F).** The most discriminative cut-offs are highlighted by a dotted square.

**
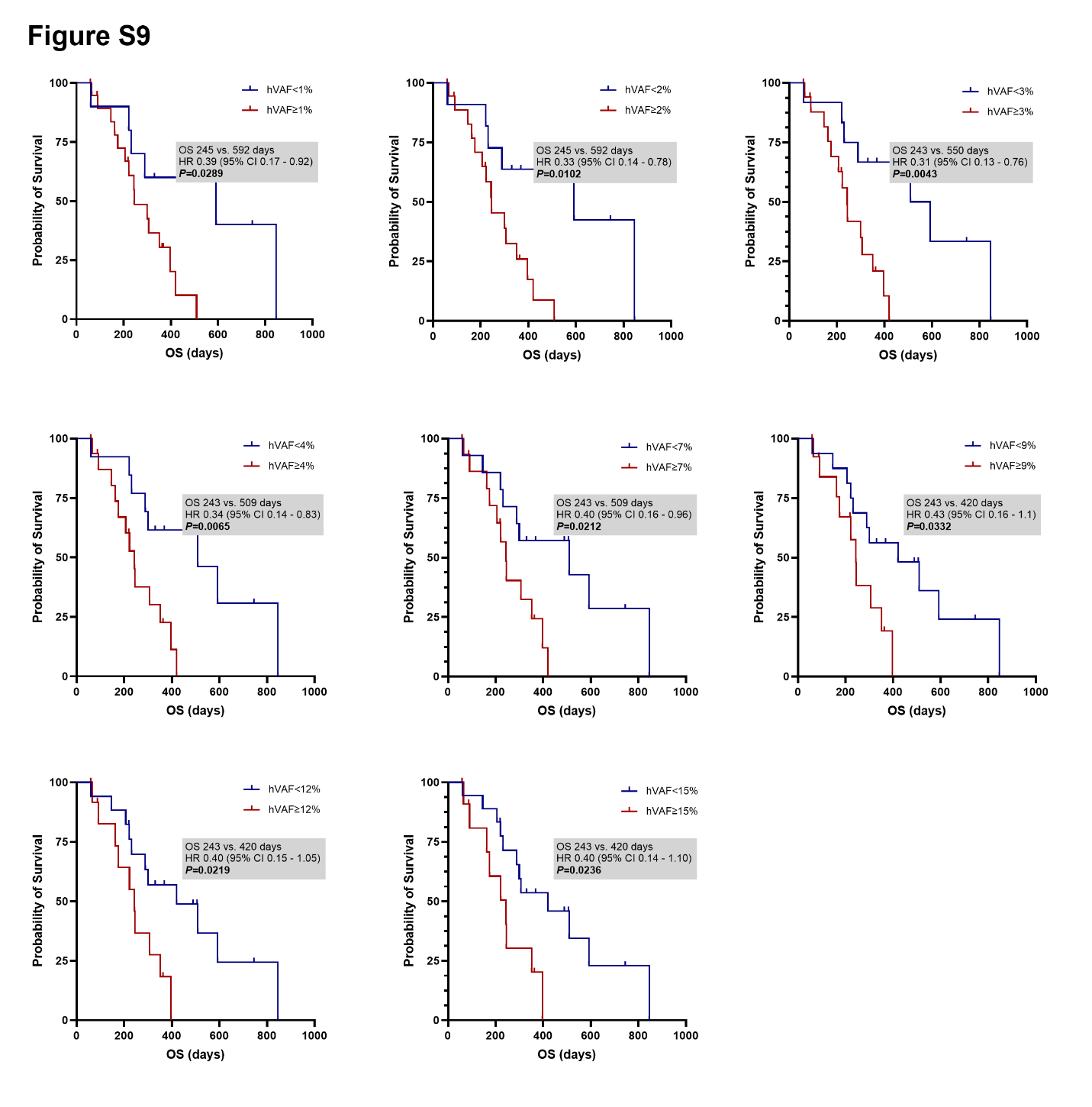
**

Figure S9. Kaplan-Meier curves of OS calculated from the first blood sample. Kaplan-Meier stratified at baseline (BL) by various ctDNA levels assessed as highest variant allele frequency (hVAF) (1%-10%, 12%, and 15%). KM curves for cut-offs that revealed the same outcomes (e.g. 4%, 5% and 6% or 7% and 8%) are shown only once.


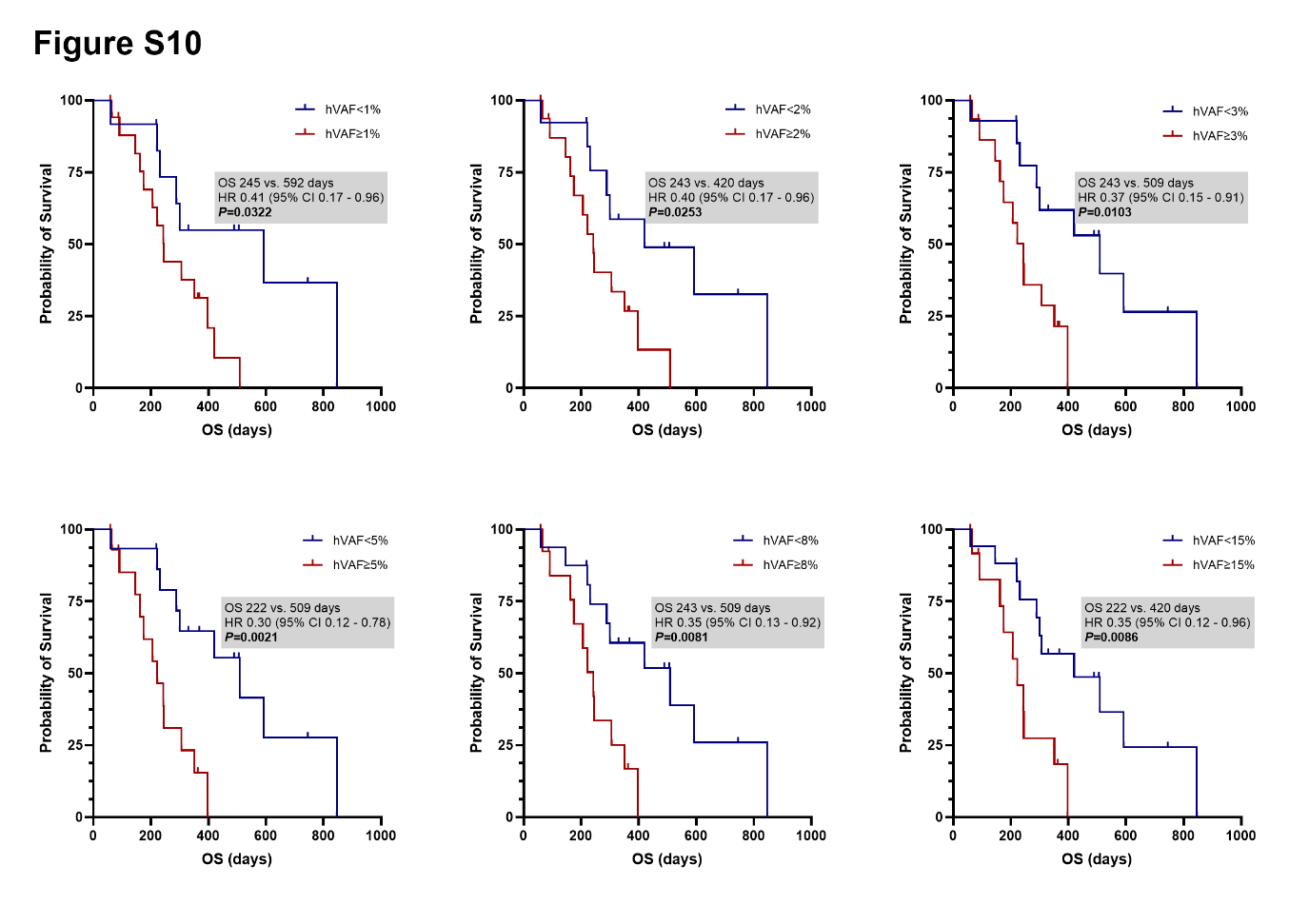


Figure S10. Kaplan-Meier curves of OS calculated from the first blood sample.

Kaplan-Meier (KM) curves stratified at the firs follow-up (FU1) by various ctDNA levels assessed as highest variant allele frequency (hVAF) (1%-10%, 12% and 15%). KM curves for cut-offs that revealed the same outcomes (e.g. 4%-7% or 8%-12%) are shown only once.


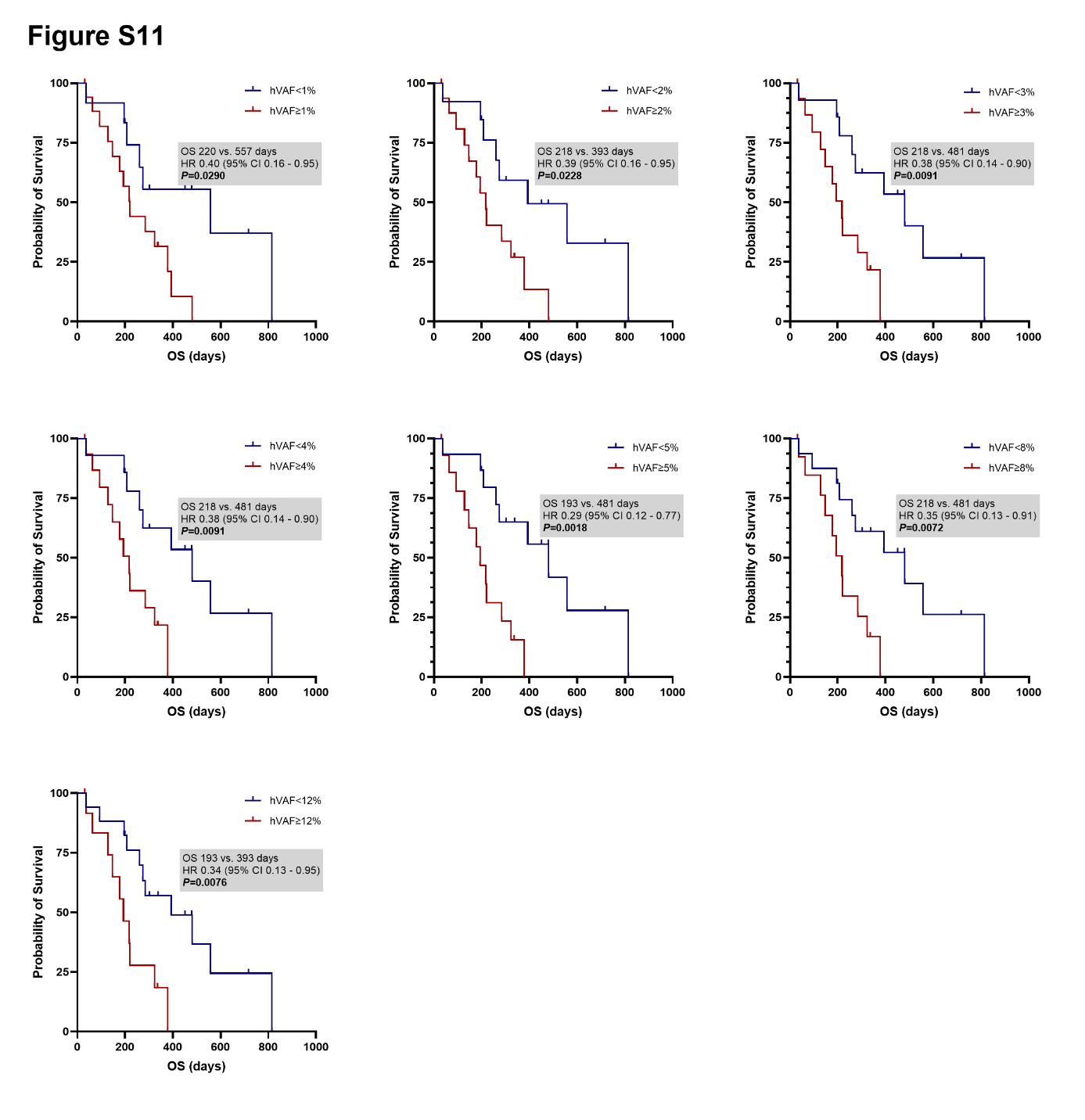


Figure S11. Kaplan-Meier curves of OS calculated from the first follow-up sample.

Kaplan-Meier (KM) stratified at the first follow-up (FU1) by various ctDNA levels assessed as highest variant allele frequency (hVAF) (1%-10%, 12% and 15%). KM curves for cut-offs that revealed the same outcomes (e.g. 4%-7% or 9%-12%) are shown only once.


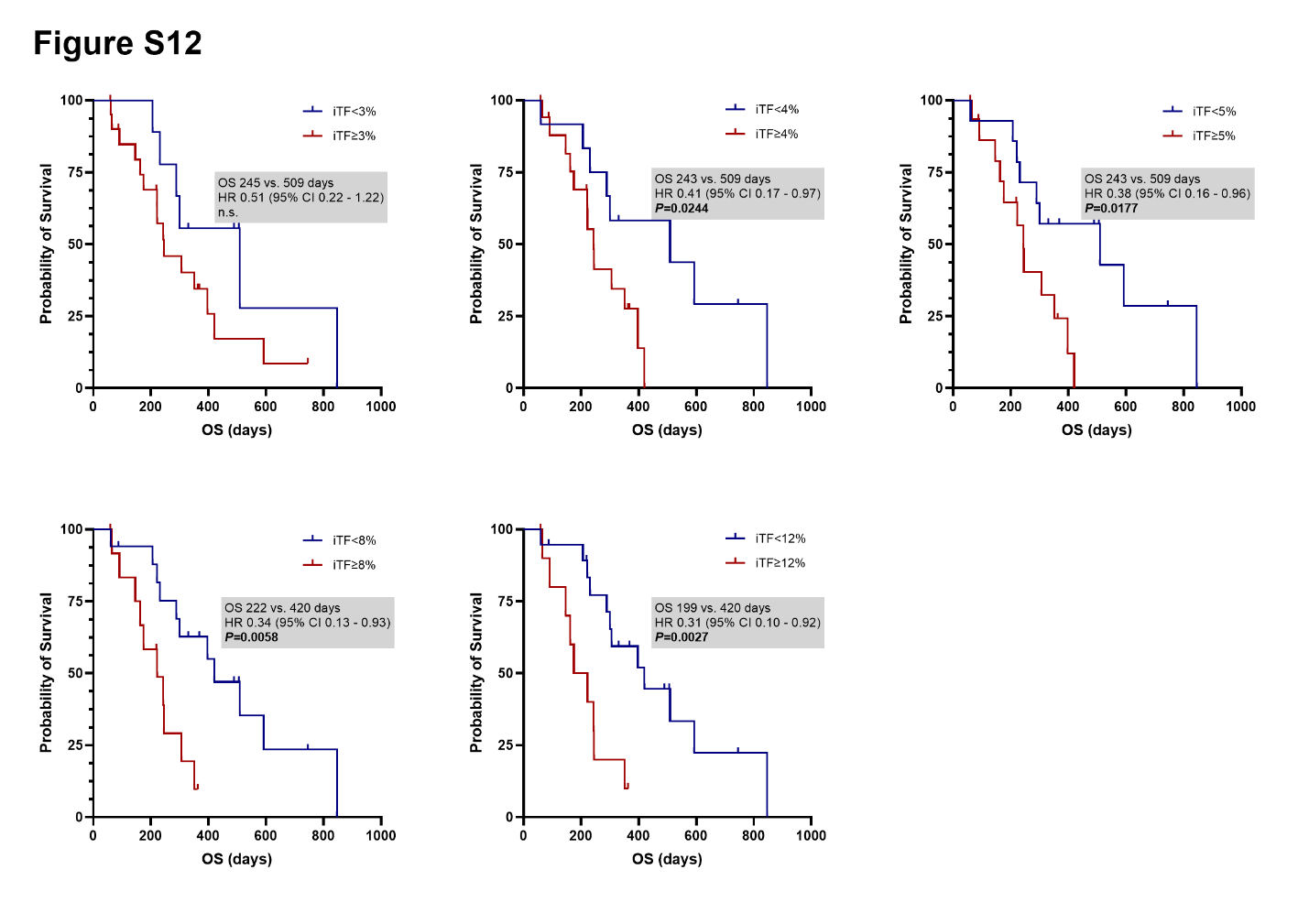


Figure S12. Kaplan-Meier curves of OS calculated from the first blood sample. Kaplan-Meier stratified at baseline (BL) by various ctDNA levels assessed as ichorCNA tumor fraction (iTF) (1%-10%, 12%, and 15%). KM curves for cut-offs that revealed the same outcomes (e.g. 5%-7%, 8%-10% and 12%, 15%) are shown only once.


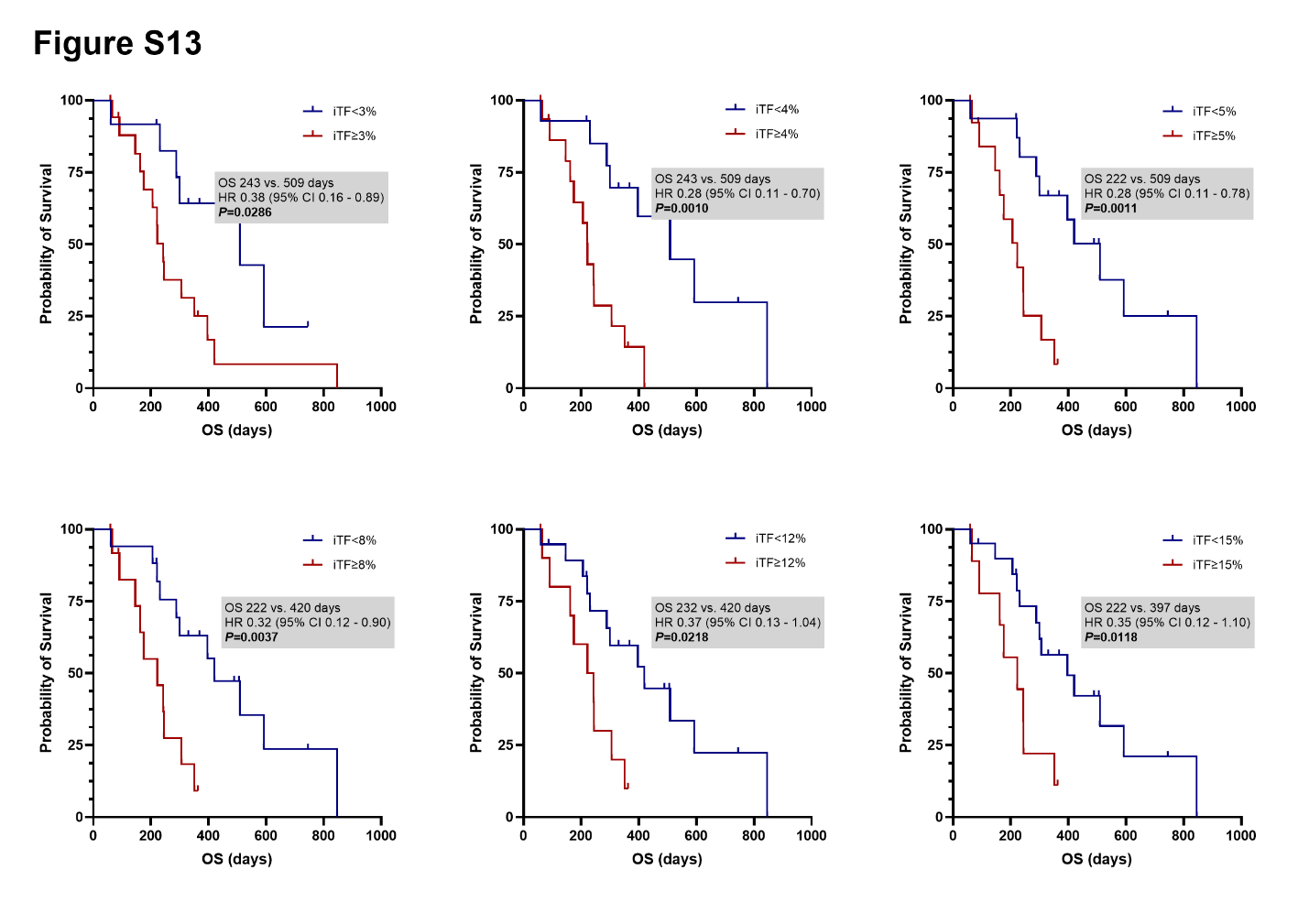


Figure S13. Kaplan-Meier curves of OS calculated from the first blood sample. Kaplan-Meier stratified at the fist follow-up (FU1) by various ctDNA levels assessed as ichorCNA tumor fraction (iTF) (1%-10%, 12%, and 15%). KM curves for cut-offs that revealed the same outcomes (e.g. 5%-7% or 8%-10%) are shown only once.


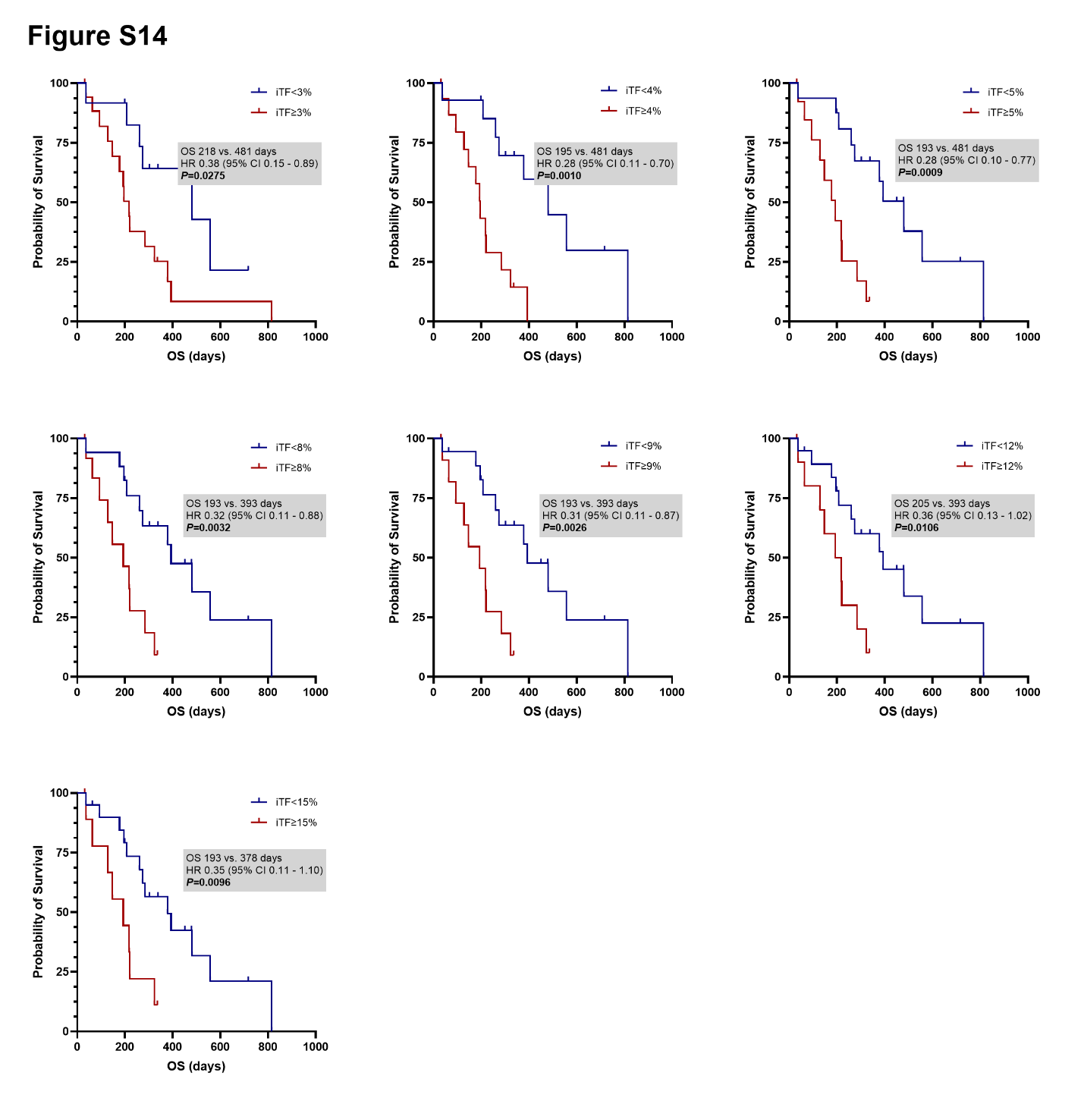


Figure S14. Kaplan-Meier curves of OS calculated from the first follow-up sample. Kaplan-Meier curves stratified at the fist follow-up (FU1) by various ctDNA levels assessed as ichorCNA tumor fraction (1%-10%, 12%, and 15%). KM curves for cut-offs that revealed the same outcomes (e.g. 5%-8% or 9%-12%) are shown only once.

Table S1. Gene list and types of detectable alterations for the AVENIO Expanded Panel

| ***Gene*** | ***SNV^1^*** | ***InDel^2^*** | ***Fusion^3^*** | ***CNV^4^*** |  | ***Gene*** | ***SNV^1^*** | ***InDel^2^*** | ***Fusion^3^*** | ***CNV^4^*** |
| --- | --- | --- | --- | --- | --- | --- | --- | --- | --- | --- |
| *ABL1* | ***x*** |  |  |  |  | *JAK3* | ***x*** |  |  |  |
| *AKT1* | ***x*** |  |  |  |  | *KDR* | ***x*** |  |  |  |
| *AKT2* | ***x*** |  |  |  |  | *KEAP1* | ***x*** |  |  |  |
| *ALK* | ***x*** | ***x*** | ***x*** |  |  | *KIT* | ***x*** | ***x*** |  |  |
| *APC* | ***x*** | ***x*** |  |  |  | *KRAS* | ***x*** |  |  |  |
| *AR* | ***x*** |  |  |  |  | *MAP2K1* | ***x*** |  |  |  |
| *ARAF* | ***x*** |  |  |  |  | *MAP2K2* | ***x*** |  |  |  |
| *BRAF* | ***x*** | ***x*** |  |  |  | *MET* | ***x*** | ***x*** |  | ***x*** |
| *BRCA1* | ***x*** |  |  |  |  | *MLH1* | ***x*** |  |  |  |
| *BRCA2* | ***x*** |  |  |  |  | *MSH2* | ***x*** |  |  |  |
| *CCND1* | ***x*** |  |  |  |  | *MSH6* | ***x*** |  |  |  |
| *CCND2* | ***x*** |  |  |  |  | *MTOR* | ***x*** |  |  |  |
| *CCND3* | ***x*** |  |  |  |  | *NF2* | ***x*** |  |  |  |
| *CD274* | ***x*** |  |  |  |  | *NFE2L2* | ***x*** |  |  |  |
| *CDK4* | ***x*** |  |  |  |  | *NRAS* | ***x*** |  |  |  |
| *CDK6* | ***x*** |  |  |  |  | *NTRK1* | ***x*** |  | ***x*** |  |
| *CDKN2A* | ***x*** |  |  |  |  | *PDCD1LG2* | ***x*** |  |  |  |
| *CSF1R* | ***x*** |  |  |  |  | *PDGFRA* | ***x*** |  |  |  |
| *CTNNB1* | ***x*** | ***x*** |  |  |  | *PDGFRB* | ***x*** |  |  |  |
| *DDR2* | ***x*** |  |  |  |  | *PIK3CA* | ***x*** | ***x*** |  |  |
| *DPYD* | ***x*** |  |  |  |  | *PIK3R1* | ***x*** |  |  |  |
| *EGFR* | ***x*** | ***x*** |  | ***x*** |  | *PMS2* | ***x*** |  |  |  |
| *ERBB2* | ***x*** | ***x*** |  | ***x*** |  | *PTCH1* | ***x*** |  |  |  |
| *ESR1* | ***x*** |  |  |  |  | *PTEN* | ***x*** | ***x*** |  |  |
| *EZH2* | ***x*** |  |  |  |  | *RAF1* | ***x*** |  |  |  |
| *FBXW7* | ***x*** |  |  |  |  | *RB1* | ***x*** |  |  |  |
| *FGFR1* | ***x*** |  |  |  |  | *RET* | ***x*** |  | ***x*** |  |
| *FGFR2* | ***x*** |  | ***x*** |  |  | *RNF43* | ***x*** |  |  |  |
| *FGFR3* | ***x*** |  | ***x*** |  |  | *ROS1* | ***x*** |  | ***x*** |  |
| *FLT1* | ***x*** |  |  |  |  | *SMAD4* | ***x*** |  |  |  |
| *FLT3* | ***x*** |  |  |  |  | *SMO* | ***x*** |  |  |  |
| *FLT4* | ***x*** |  |  |  |  | *STK11* | ***x*** |  |  |  |
| *GATA3* | ***x*** |  |  |  |  | *TERT* | ***x*** |  |  |  |
| *GNA11* | ***x*** |  |  |  |  | *TP53* | ***x*** | ***x*** |  |  |
| *GNAQ* | ***x*** |  |  |  |  | *TSC1* | ***x*** | ***x*** |  |  |
| *GNAS* | ***x*** |  |  |  |  | *TSC2* | ***x*** |  |  |  |
| *IDH1* |  |  |  |  |  | *UGT1A1* | ***x*** |  |  |  |
| *IDH2* | ***x*** |  |  |  |  | *VHL* | ***x*** |  |  |  |
| *JAK2* | ***x*** |  |  |  |  |  |  |  |  |  |

^1^SNV is single nucleotide variants

^2^InDel is insertion/deletion variants

^3^Fusion is fusion products resulting from rearranged genes

^4^CNV is copy number variations

Information from <https://sequencing.roche.com/en/products-solutions/products/ngs-oncology-assays/ctdna-analysis-kits.html>

Table S2. The sequencing coverage and quality statistics of the enriched data

| **Sample ID** | **Total no. of**  **sequenced reads** | **Total no. of uniquely mapped non‐ duplicate reads** | **Total no. of**  **covered targeted bases [kb]** | **Median coverage (and range) per targeted base** | **Percentage of targeted bases with coverage ≥200** |
| --- | --- | --- | --- | --- | --- |
| 2018_4_1 | 32361016 | 20106196 | 162.1 | 4553 (176-10763) | 100,00 |
| 2018_4_2 | 21264550 | 14707379 | 162.1 | 3434 (159-7044) | 100,00 |
| 2018_4_3 | 41880860 | 21987483 | 162.1 | 4372 (252-10286) | 100,00 |
| 2018_4_4 | 31779808 | 18780024 | 162.1 | 4083 (246-11327) | 100,00 |
| 2019_10_1 | 30759172 | 11390894 | 162.1 | 2740 (109-4751) | 100,00 |
| 2019_11_1 | 30680501 | 11012754 | 162.1 | 2358 (103-3832) | 100,00 |
| 2019_12_1 | 48706356 | 26180944 | 162.1 | 5818 (312-13478) | 100,00 |
| 2019_13_1 | 38056498 | 11605137 | 162.1 | 2277 (29-4183) | 100,00 |
| 2019_15_1 | 32681717 | 11037191 | 162.1 | 2092 (29-3677) | 100,00 |
| 2019_16_1 | 45402570 | 22763728 | 162.1 | 5314 (208-10180) | 100,00 |
| 2019_17_1 | 44052249 | 21476556 | 162.1 | 4768 (288-10599) | 100,00 |
| 2019_18_1 | 33210474 | 33938369 | 162.1 | 4576 (136-8452) | 100,00 |
| 2019_19_1 | 43998844 | 16306014 | 162.1 | 3983 (49-10845) | 100,00 |
| 2019_2_1 | 43378811 | 18910046 | 162.1 | 3907 (118-7878) | 100,00 |
| 2019_20_1 | 37791637 | 13633988 | 162.1 | 2867 (34-4614) | 100,00 |
| 2019_21_1 | 52278578 | 22042784 | 162.1 | 5195 (62-12356) | 100,00 |
| 2019_22_1 | 49455971 | 19984579 | 162.1 | 4734 (0-11751) | 100,00 |
| 2019_3_1 | 39917787 | 22337046 | 162.1 | 5070 (160-8037) | 100,00 |
| 2019_4_1 | 51365605 | 24301459 | 162.1 | 5320 (2-9731) | 100,00 |
| 2019_5_1 | 46134037 | 21904104 | 162.1 | 4645 (9-7440) | 100,00 |
| 2019_6_1 | 53094279 | 19473872 | 162.1 | 3678 (43-5946) | 100,00 |
| 2019_7_1 | 46688486 | 17841155 | 162.1 | 3756 (65-6179) | 100,00 |
| 2019_8_1 | 49527211 | 15366606 | 162.1 | 3039 (27-7687) | 100,00 |
| 2019_9_1 | 29190521 | 15781247 | 162.1 | 3965 (167-7452) | 100,00 |
| 2019_10_2 | 20500823 | 8326536 | 162.1 | 1853 (67-3161) | 100,00 |
| 2019_10_3 | 37961498 | 12450176 | 162.1 | 1647 (41-3118) | 100,00 |
| 2019_10_4 | 38273294 | 10578648 | 162.1 | 1980 (67-3740) | 100,00 |
| 2019_11_2 | 40768425 | 6163221 | 162.1 | 974 (31-2296) | 100,00 |
| 2019_11_3 | 40128138 | 20065731 | 162.1 | 3300 (155-5177) | 100,00 |
| 2019_12_2 | 31224082 | 17921618 | 162.1 | 3749 (188-8783) | 100,00 |
| 2019_12_3 | 32422900 | 22781996 | 162.1 | 3534 (211-7716) | 100,00 |
| 2019_13_2 | 29330862 | 6408192 | 162.1 | 1027 (8-2196) | 100,00 |
| 2019_13_3 | 36595703 | 10623187 | 162.1 | 1891 (20-3462) | 100,00 |
| 2019_15_2 | 30307027 | 7959693 | 162.1 | 1364 (24-2751) | 100,00 |
| 2019_16_2 | 41925574 | 21111850 | 162.1 | 4640 (163-11311) | 100,00 |
| 2019_16_3 | 43139999 | 19002426 | 162.1 | 3992 (62-10180) | 100,00 |
| 2019_16_4 | 43542571 | 22735553 | 162.1 | 4788 (353-10116) | 100,00 |
| 2019_17_2 | 42966077 | 19049340 | 162.1 | 3934 (18-8782) | 100,00 |
| 2019_17_3 | 41034838 | 20326040 | 162.1 | 3996 (5-6251) | 100,00 |
| 2019_17_4 | 45296388 | 20759865 | 162.1 | 4559 (89-8287) | 100,00 |
| 2019_18_2 | 37721370 | 11875667 | 162.1 | 2188 (45-4602) | 100,00 |
| 2019_18_3 | 37116119 | 11587666 | 162.1 | 2110 (43-3591) | 100,00 |
| 2019_18_4 | 46505291 | 20459787 | 162.1 | 4411 (113-8033) | 100,00 |
| 2019_19_2 | 38428649 | 16238610 | 162.1 | 3706 (61-8813) | 100,00 |
| 2019_19_3 | 34484057 | 10138133 | 162.1 | 1723 (0-3883) | 100,00 |
| 2019_19_4 | 46994487 | 17153533 | 162.1 | 3600 (1-6692) | 100,00 |
| 2019_2_2 | 21952437 | 11847832 | 162.1 | 2616 (79-5526) | 100,00 |
| 2019_2_3 | 35757137 | 20177441 | 162.1 | 3877 (110-7555) | 100,00 |
| 2019_20_2 | 38228300 | 11373234 | 162.1 | 2085 (19-3926) | 100,00 |
| 2019_20_3 | 45570518 | 10634546 | 162.1 | 1286 (10-2961) | 100,00 |
| 2019_20_4 | 44647400 | 9911200 | 162.1 | 1118 (0-2659) | 100,00 |
| 2019_21_2 | 36258411 | 14916882 | 162.1 | 3132 (142-6637) | 100,00 |
| 2019_22_2 | 34394421 | 10329745 | 162.1 | 1883 (16-4483) | 100,00 |
| 2019_22_3 | 59543485 | 23394135 | 162.1 | 4046.5 (0-8133) | 100,00 |
| 2019_22_4 | 47715349 | 18928655 | 162.1 | 3824 (0-7717) | 100,00 |
| 2019_3_2 | 27155559 | 16883722 | 162.1 | 3643 (107-5584) | 100,00 |
| 2019_3_3 | 17204812 | 11162405 | 162.1 | 2078 (95-3314) | 100,00 |
| 2019_3_4 | 28966213 | 15309292 | 162.1 | 3371 (119-5217) | 100,00 |
| 2019_4_2 | 24425001 | 14860408 | 162.1 | 3389 (37-6753) | 100,00 |
| 2019_4_3 | 40930108 | 20531757 | 162.1 | 3987 (68-8770) | 100,00 |
| 2019_4_4 | 32893738 | 18112778 | 162.1 | 4137 (48-9852) | 100,00 |
| 2019_5_2 | 21891985 | 14056383 | 162.1 | 3399 (47-5985) | 100,00 |
| 2019_6_2 | 20338254 | 6747878 | 162.1 | 1276 (17-2214) | 100,00 |
| 2019_6_3 | 38224808 | 10536119 | 162.1 | 1656 (17-3047) | 100,00 |
| 2019_6_4 | 22518506 | 7201581 | 162.1 | 1399 (11-2337) | 100,00 |
| 2019_7_2 | 18283981 | 8984328 | 162.1 | 2054 (34-3448) | 100,00 |
| 2019_7_3 | 43857602 | 11946509 | 162.1 | 1836 (24-3232) | 100,00 |
| 2019_7_4 | 28441692 | 6793172 | 162.1 | 1270 (17-2475) | 100,00 |
| 2019_8_2 | 19544407 | 8515256 | 162.1 | 1743 (20-3789) | 100,00 |
| 2019_8_3 | 33399619 | 11423561 | 162.1 | 1877 (31-4090) | 100,00 |
| 2019_8_4 | 39114247 | 8575939 | 162.1 | 1526 (25-4289) | 100,00 |
| 2019_9_2 | 23173561 | 14588867 | 162.1 | 3600 (134-7156) | 100,00 |
| 2019_9_3 | 35836664 | 23092594 | 162.1 | 4164 (207-7949) | 100,00 |
| 2019_9_4 | 35292182 | 21303598 | 162.1 | 4374 (256-8433) | 100,00 |
| 2020_1_1 | 42864060 | 17779976 | 162.1 | 4229 (0-8398) | 100,00 |
| 2020_2_1 | 54872731 | 23868003 | 162.1 | 5628 (0-13362) | 100,00 |
| 2020_3_1 | 32694772 | 11356843 | 162.1 | 2368 (17-8851) | 100,00 |
| 2020_4_1 | 43494676 | 23453880 | 162.1 | 5136 (65-9461) | 100,00 |
| 2020_5_1 | 30440199 | 19118276 | 162.1 | 3748 (192-6167) | 100,00 |
| 2020_6_1 | 30788739 | 8940918 | 162.1 | 1439 (0-2799) | 100,00 |
| 2020_7_1 | 27746644 | 14566974 | 162.1 | 2593 (56-5467) | 100,00 |
| 2020_8_1 | 36832503 | 18072541 | 162.1 | 3215 (132-8915) | 100,00 |
| 2020_9_1 | 45937809 | 27951967 | 162.1 | 5925 (100-11169) | 100,00 |
| 2020_1_2 | 36883242 | 19548037 | 162.1 | 4430 (1-8705) | 100,00 |
| 2020_1_3 | 47362199 | 11941905 | 162.1 | 1895 (0-3648) | 100,00 |
| 2020_1_4 | 33324437 | 8830262 | 162.1 | 1726 (0-3079) | 100,00 |
| 2020_2_2 | 39235591 | 19250456 | 162.1 | 3825 (28-6809) | 100,00 |
| 2020_2_3 | 38955825 | 17961502 | 162.1 | 3111 (2-5235) | 100,00 |
| 2020_2_4 | 35852055 | 17163691 | 162.1 | 3791 (2-6281) | 100,00 |
| 2020_3_2 | 40605846 | 10217622 | 162.1 | 1608 (17-4966) | 100,00 |
| 2020_3_3 | 32911483 | 18399279 | 162.1 | 3214 (52-5738) | 100,00 |
| 2020_3_4 | 31999511 | 13139961 | 162.1 | 2397 (52-4785) | 100,00 |
| 2020_4_2 | 44252560 | 16237615 | 162.1 | 2753 (131-5383) | 100,00 |
| 2020_4_3 | 30865541 | 22727889 | 162.1 | 4180 (240-8615) | 100,00 |
| 2020_4_4 | 35319849 | 19868780 | 162.1 | 3999 (284-7647) | 100,00 |
| 2020_5_2 | 47348386 | 15413099 | 162.1 | 2248 (71-4768) | 100,00 |
| 2020_5_3 | 50169931 | 14655810 | 162.1 | 1747 (1-4133) | 100,00 |
| 2020_5_4 | 33973768 | 4953300 | 162.1 | 801 (21-2133) | 99,99 |
| 2020_6_2 | 40546401 | 8918700 | 162.1 | 1340 (0-2587) | 100,00 |
| 2020_6_3 | 33640075 | 15936045 | 162.1 | 2713 (7-5475) | 100,00 |
| 2020_7_2 | 33016321 | 13044446 | 162.1 | 2038 (76-3410) | 100,00 |
| 2020_7_3 | 33633417 | 11204258 | 162.1 | 1370 (27-3287) | 100,00 |
| 2020_8_2 | 32306552 | 15431745 | 162.1 | 2908 (232-5848) | 100,00 |
| 2020_8_3 | 25338895 | 11396075 | 162.1 | 1857 (78-3198) | 100,00 |
| 2020_9_2 | 16463493 | 12877225 | 162.1 | 2887 (100-7116) | 100,00 |
| 2020_9_3 | 31464660 | 21279494 | 162.1 | 4505 (149-9028) | 100,00 |
| 2020_9_4 | 42769355 | 22298669 | 162.1 | 4760 (135-9091) | 100,00 |

Table S3. The sequencing coverage and quality statistics of the shallow whole genome sequencing

| **Sample  ID** | **Total no. of**  **sequenced reads** | **Total no. of uniquely assigned unduplicated reads** | **Total no. of**  **bases covered** | **Median coverage**  **(and range) per base** | **Percentage of target bases with coverage ≥1** |
| --- | --- | --- | --- | --- | --- |
| 2018_04_1 | 4583487 | 3391632 | 232944292 | 0 | 7,51 |
| 2018_04_2 | 3319404 | 1748278 | 122830812 | 0 | 3,96 |
| 2018_04_3 | 4190719 | 2431578 | 169047455 | 0 | 5,45 |
| 2018_04_4 | 4196059 | 2472036 | 171528886 | 0 | 5,53 |
| 2019_02_1 | 3899179 | 2720939 | 188588721 | 0 | 6,08 |
| 2019_02_2 | 3471821 | 2237848 | 156330124 | 0 | 5,04 |
| 2019_02_3 | 3938129 | 2904832 | 200685695 | 0 | 6,47 |
| 2019_03_1 | 3836423 | 2781915 | 192621045 | 0 | 6,21 |
| 2019_03_2 | 3677840 | 2478684 | 172459422 | 0 | 5,56 |
| 2019_03_3 | 3980082 | 2642796 | 183315681 | 0 | 5,91 |
| 2019_03_4 | 3911819 | 2729583 | 189209078 | 0 | 6,10 |
| 2019_04_1 | 4759496 | 3656973 | 250004127 | 0 | 8,06 |
| 2019_04_2 | 3702761 | 2471062 | 171528886 | 0 | 5,53 |
| 2019_04_3 | 4381223 | 3174309 | 217745530 | 0 | 7,02 |
| 2019_04_4 | 4430605 | 3417685 | 233564649 | 0 | 7,53 |
| 2019_05_1 | 4430558 | 3328381 | 228911967 | 0 | 7,38 |
| 2019_05_2 | 4538291 | 3155471 | 217435351 | 0 | 7,01 |
| 2019_06_1 | 3528693 | 2647506 | 183936038 | 0 | 5,93 |
| 2019_06_2 | 2896601 | 1646903 | 116006878 | 0 | 3,74 |
| 2019_06_3 | 4125029 | 2659661 | 184556396 | 0 | 5,95 |
| 2019_06_4 | 4157709 | 1979236 | 138649931 | 0 | 4,47 |
| 2019_07_1 | 4981494 | 3565232 | 243800550 | 0 | 7,86 |
| 2019_07_2 | 3091081 | 1681256 | 118178129 | 0 | 3,81 |
| 2019_07_3 | 3558422 | 2310939 | 160982806 | 0 | 5,19 |
| 2019_07_4 | 3501847 | 2137739 | 149196011 | 0 | 4,81 |
| 2019_08_1 | 4704779 | 3359662 | 230773040 | 0 | 7,44 |
| 2019_08_2 | 2833763 | 1679459 | 117867950 | 0 | 3,80 |
| 2019_08_3 | 4069500 | 2862376 | 198204264 | 0 | 6,39 |
| 2019_08_4 | 3159277 | 2150899 | 150436726 | 0 | 4,85 |
| 2019_09_1 | 4718750 | 3231079 | 222088033 | 0 | 7,16 |
| 2019_09_2 | 3710517 | 2439235 | 169357634 | 0 | 5,46 |
| 2019_09_3 | 4858422 | 3704971 | 252485557 | 0 | 8,14 |
| 2019_09_4 | 4910762 | 3669825 | 250624484 | 0 | 8,08 |
| 2019_10_1 | 3617885 | 2291860 | 159742091 | 0 | 5,15 |
| 2019_10_2 | 2821565 | 1698840 | 119418845 | 0 | 3,85 |
| 2019_10_3 | 3840004 | 2626480 | 182074966 | 0 | 5,87 |
| 2019_10_4 | 4860024 | 3514559 | 241008941 | 0 | 7,77 |
| 2019_11_1 | 3024693 | 2132316 | 149196011 | 0 | 4,81 |
| 2019_11_2 | 3398257 | 2225115 | 155399587 | 0 | 5,01 |
| 2019_11_3 | 4969392 | 3587692 | 245661623 | 0 | 7,92 |
| 2019_12_1 | 5885556 | 4445588 | 299632737 | 0 | 9,66 |
| 2019_12_2 | 4137766 | 2967275 | 204407840 | 0 | 6,59 |
| 2019_12_3 | 3931316 | 2954277 | 203787483 | 0 | 6,57 |
| 2019_13_1 | 4096088 | 2600289 | 180524071 | 0 | 5,82 |
| 2019_13_2 | 3655854 | 2377089 | 165325309 | 0 | 5,33 |
| 2019_13_3 | 4049598 | 2749729 | 190449794 | 0 | 6,14 |
| 2019_15_1 | 3756038 | 2465658 | 171528886 | 0 | 5,53 |
| 2019_15_2 | 3516216 | 2469425 | 171839065 | 0 | 5,54 |
| 2019_16_1 | 5972863 | 4535486 | 305836314 | 0 | 9,86 |
| 2019_16_2 | 5054696 | 3331417 | 227981430 | 0 | 7,35 |
| 2019_16_3 | 4738674 | 3323152 | 227671252 | 0 | 7,34 |
| 2019_16_4 | 5247168 | 4012129 | 271716644 | 0 | 8,76 |
| 2019_17_1 | 6037151 | 4201126 | 284744154 | 0 | 9,18 |
| 2019_17_2 | 5205886 | 4058487 | 275748968 | 0 | 8,89 |
| 2019_17_3 | 4750427 | 3568009 | 244110729 | 0 | 7,87 |
| 2019_17_4 | 4632291 | 3667142 | 250934663 | 0 | 8,09 |
| 2019_18_1 | 3362921 | 2534211 | 175871389 | 0 | 5,67 |
| 2019_18_2 | 3984068 | 2303913 | 158191197 | 0 | 5,10 |
| 2019_18_3 | 3297094 | 2022829 | 141441541 | 0 | 4,56 |
| 2019_18_4 | 4564235 | 3506710 | 239768226 | 0 | 7,73 |
| 2019_19_1 | 4869645 | 3542196 | 242870014 | 0 | 7,83 |
| 2019_19_2 | 4305667 | 3105517 | 214333563 | 0 | 6,91 |
| 2019_19_3 | 4440742 | 1913662 | 133997249 | 0 | 4,32 |
| 2019_19_4 | 4606431 | 3614862 | 246902338 | 0 | 7,96 |
| 2019_20_1 | 3337602 | 2375159 | 165635488 | 0 | 5,34 |
| 2019_20_2 | 4758098 | 3542952 | 242870014 | 0 | 7,83 |
| 2019_20_3 | 3204012 | 2144757 | 149816369 | 0 | 4,83 |
| 2019_20_4 | 3199575 | 2064897 | 144543329 | 0 | 4,66 |
| 2019_21_1 | 4707146 | 3517041 | 240698762 | 0 | 7,76 |
| 2019_21_2 | 3906321 | 2762899 | 191070151 | 0 | 6,16 |
| 2019_22_1 | 4321339 | 2986917 | 206268913 | 0 | 6,65 |
| 2019_22_2 | 3186609 | 2051363 | 143302613 | 0 | 4,62 |
| 2019_22_3 | 4264341 | 3118185 | 214953920 | 0 | 6,93 |
| 2019_22_4 | 4767667 | 3625627 | 247832875 | 0 | 7,99 |
| 2020_1_1 | 3708244 | 2816943 | 194792297 | 0 | 6,28 |
| 2020_1_2 | 3656676 | 2782652 | 191690509 | 0 | 6,18 |
| 2020_1_3 | 3438982 | 2286993 | 159431912 | 0 | 5,14 |
| 2020_1_4 | 3496293 | 2257109 | 157260660 | 0 | 5,07 |
| 2020_2_1 | 5123853 | 3937823 | 267994498 | 0 | 8,64 |
| 2020_2_2 | 4086708 | 3157851 | 217435351 | 0 | 7,01 |
| 2020_2_3 | 4042372 | 3162569 | 217745530 | 0 | 7,02 |
| 2020_2_4 | 4543035 | 3362365 | 229842503 | 0 | 7,41 |
| 2020_3_1 | 3549931 | 2619001 | 181764787 | 0 | 5,86 |
| 2020_3_2 | 3490932 | 2663288 | 184246217 | 0 | 5,94 |
| 2020_3_3 | 3653705 | 2660044 | 184246217 | 0 | 5,94 |
| 2020_3_4 | 3524014 | 2431035 | 168427098 | 0 | 5,43 |
| 2020_4_1 | 8986697 | 6892637 | 450069463 | 0 | 14,51 |
| 2020_4_2 | 4087457 | 2883873 | 198514443 | 0 | 6,40 |
| 2020_4_3 | 4534008 | 3308078 | 226120358 | 0 | 7,29 |
| 2020_4_4 | 5950983 | 4459771 | 299942916 | 0 | 9,67 |
| 2020_5_1 | 3209022 | 2365912 | 165015131 | 0 | 5,32 |
| 2020_5_2 | 3571098 | 2677516 | 185176754 | 0 | 5,97 |
| 2020_5_3 | 4402355 | 3197263 | 218986245 | 0 | 7,06 |
| 2020_5_4 | 3335001 | 1986244 | 138960110 | 0 | 4,48 |
| 2020_6_1 | 2109742 | 1405367 | 99257221 | 0 | 3,20 |
| 2020_6_2 | 3103314 | 1681907 | 118178129 | 0 | 3,81 |
| 2020_6_3 | 4115846 | 2952375 | 203167125 | 0 | 6,55 |
| 2020_7_1 | 3538308 | 2465709 | 171528886 | 0 | 5,53 |
| 2020_7_2 | 2809805 | 1610561 | 113215268 | 0 | 3,65 |
| 2020_7_3 | 3662218 | 2365964 | 164704952 | 0 | 5,31 |
| 2020_8_1 | 4445170 | 3308332 | 227361073 | 0 | 7,33 |
| 2020_8_2 | 3023481 | 2079298 | 144853508 | 0 | 4,67 |
| 2020_8_3 | 3581707 | 2622756 | 181764787 | 0 | 5,86 |
| 2020_9_1 | 5682627 | 4470223 | 301493810 | 0 | 9,72 |
| 2020_9_2 | 4137321 | 2820701 | 193861761 | 0 | 6,25 |
| 2020_9_3 | 3974925 | 2874522 | 197583906 | 0 | 6,37 |
| 2020_9_4 | 4521620 | 3245982 | 221777854 | 0 | 7,15 |

Table S4. Overview of detected mutations and various measures of tumor fractions

(See Excel file)

Table S5. Correlation of tumor levels as hVAF (highest variant allele frequency) and blood makers

| **Blood marker** | **Correlation coefficient** | ***P-*value** |
| --- | --- | --- |
| CEA | 0.358 | 0.0567 |
| **CA19-9** | ***0.414*** | ***0.0398*** |
| Erythrocytes | 0.234 | 0.2407 |
| Hemoglobin | 0.125 | 0.5335 |
| Leukocytes | 0.290 | 0.1418 |
| Lymphocytes | 0.020 | 0.9374 |
| ***Thrombocytes*** | ***0.432*** | ***0.0243*** |
| ***Neutrophiles*** | ***0.545*** | ***0.0195*** |
| ***C-reactive protein (CRP)*** | ***0.421*** | ***0.0258*** |
| ***Lactate dehydrogenase (LDH)*** | ***0.587*** | ***0.0010*** |
| Alkaline phosphatase (ALP) | 0.088 | 0.6551 |
| Bilirubins | 0.192 | 0.3266 |
| Aspartate aminotransferase (AST/GOT) | 0.020 | 0.9148 |
| Alanine aminotransferase (ALT/GPT) | 0.085 | 0.6709 |
| Albumins | 0.243 | 0.2126 |
| Creatinine | 0.356 | 0.0632 |

Table S6. Univariate Cox regression model for OS based on ctDNA level (iTF) at FU1 and clinical variables

| **Data** | **Variable** | **Hazard ratio** | **95% CI** | **Significance** |
| --- | --- | --- | --- | --- |
| **Categorical** | **iTF ≥ 5%** | 5.198 | 1,852 - 16,76 | * |
|  | **ECOG** | 2.323 | 0.987 - 5.029 | n.s. |
|  | **Sex** | 1.006 | 0.376 - 2.470 | n.s. |
|  | **Age ≥ 70** | 1.369 | 0.533 - 3.328 | n.s. |
|  | **Meta ≥ 2** | 0.843 | 0.339 - 2.045 | n.s. |
|  | **LDH ≥ 240** | 3.103 | 1.071 - 8.784 | * |
|  | **CRP ≥ 5** | 0.000 | ND | n.s. |
|  | **CA19-9 ≥ 37** | 1.997 | 0.740 - 5.486 | n.s. |
| **Continuous** | **iTF** | 1.053 | 1,014 - 1,093 | * |
|  | **Age** | 1.000 | 0.961 - 1.041 | n.s. |
|  | **No. of metastases** | 0.807 | 0.505 - 1.216 | n.s. |
|  | **No. of prior palliative therapies** | 0.887 | 0.615 - 1.262 | n.s. |
|  | **LDH** | 1.007 | 1.003 - 1.011 | * |
|  | **CRP** | 1.079 | 0.777 - 1.425 | n.s. |
|  | **CA19-9** | 1.001 | 0.999 - 1.002 | n.s. |

Table S7. Multivariate Cox regression model for OS, including ctDNA levels (iTF at FU1) and clinical variables

| **Variable** | **Hazard ratio** | **95% CI** | **Significance** |
| --- | --- | --- | --- |
| **iTF FU1 cont.** | 1.045 | 1.008 - 1.084 | * |
| **ECOG** | 1.950 | 0.8444 - 4.283 | n.s. |
| **Met ≥ 2** | 0.9348 | 0.3394 - 2.445 | n.s. |
|  | | | |
| **Variable** | **Hazard ratio** | **95% CI** | **Significance** |
| **iTF FU1** | 1,035 | 0,9917 - 1,081 | n.s. |
| **ECOG** | 1,845 | 0,7659 - 4,244 | n.s. |
| **Met ≥ 2** | 0,9441 | 0,3349 - 2,551 | n.s. |
| **LDH > 240** | 1,480 | 0,3801 - 5,200 | n.s. |

n.s. not significant

Table S8. Multivariate Cox regression model for OS, including ctDNA levels (iTF at FU1) and clinical variables

| **Variable** | **Hazard ratio** | **95% CI** | **Significance** |
| --- | --- | --- | --- |
| **ECOG** | 2,666 | 1,149 to 6,005 | * |
| **Met ≥ 2** | 0,8647 | 0,3153 to 2,233 | n.s. |
| **iTF ≥ 5%** | 5,796 | 1,996 to 19,33 | * |
|  | | | |
| **Variable** | **Hazard ratio** | **95% CI** | **Significance** |
| **ECOG** | 2.461 | 0.9937 - 5.936 | n.s. |
| **Met ≥ 2** | 0.9008 | 0.3212 - 2.418 | n.s. |
| **LDH > 240** | 1.235 | 0.3456 - 4.114 | n.s. |
| **iTF ≥ 5%** | 4.898 | 1.496 - 17.71 | * |

n.s. not significant
